# Correction to: Serum peptidome based biomarkers searching for monitoring minimal residual disease in adult acute lymphocytic leukemia

**DOI:** 10.1186/s12953-020-00166-4

**Published:** 2020-10-24

**Authors:** Ju Bai, Aili He, Chen Huang, Juan Yang, Wanggang Zhang, Jianli Wang, Yun Yang, Pengyu Zhang, Yang Zhang, Fuling Zhou

**Affiliations:** 1 Department of Hematology, Second Affiliated Hospital, Medical School of Xi’an Jiaotong University, Xi’an, 710004 Shaanxi Province China; 2 Department of Genetics and Molecular Biology, Medical school of Xi’an Jiaotong University/Key Laboratory of Environment and Disease-Related Gene, Ministry of Education, Xi’an, 710061 Shaanxi China

**Correction to: Proteome Sci (2014) 12:49**

**https://doi.org/10.1186/s12953-014-0049-y**

Following publication of the original article [1], the authors reported an error in Fig. 9, the western-blot bands of PF4 were not correct.

The correct Fig. 9 is shown below.

**Fig. 9:**
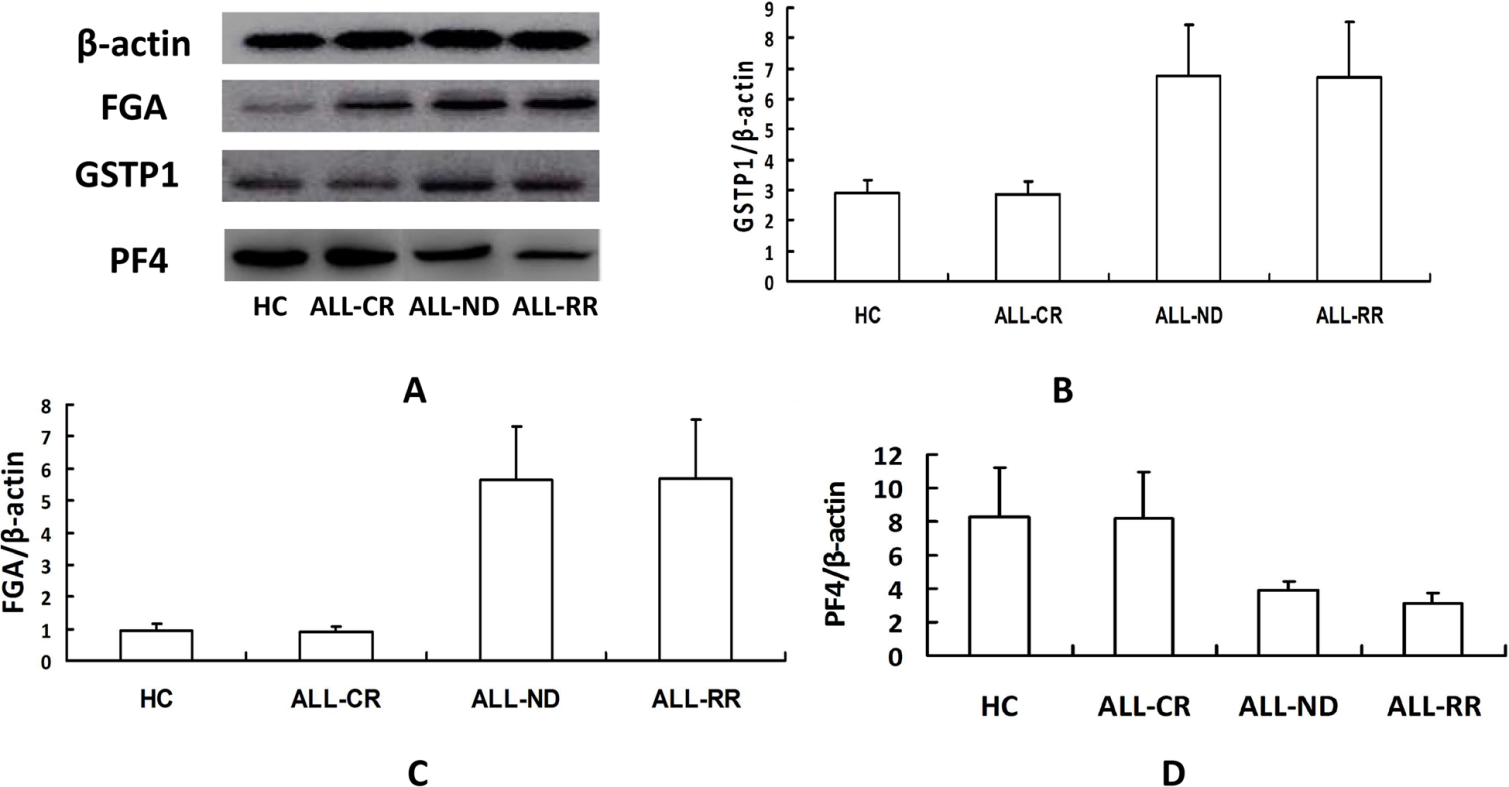
Validation of proteins by immunoblotting. **a** Levels of the FGA and GSTP1 protein differ among the leukemia cells in distinct ALL groups and normal cells. Weak PF4 immunoreactive bands are seen in newly diagnosed and refractory & relapsed ALL cases. **b** Densitometry comparison of FGA protein relative to β-actin as determined by western blot analysis in figure **a**. **c** Densitometry comparison of GSTP1 protein relative to β-actin as determined by western blot analysis in figure **a**. **d** Densitometry comparison of PF4 protein relative to β-actin as determined by western blot analysis in figure **a**. (FGA: fibrinogen alpha chain; GSTP1: glutathione S-transferase P1; PF4: platelet factor 4; ALL- CR: ALL complete remission; ALL-ND: ALL newly diagnosed; ALL-RR: ALL refractory & relapsed)
