# Supplementary figures and images for: Serum peptidome based biomarkers searching for monitoring minimal residual disease in adult acute lymphocytic leukemia
Source: Proteome Sci. 2014 Sep 16;12:49. doi: 10.1186/s12953-014-0049-y (PMC4195909; doi:10.1186/s12953-014-0049-y)

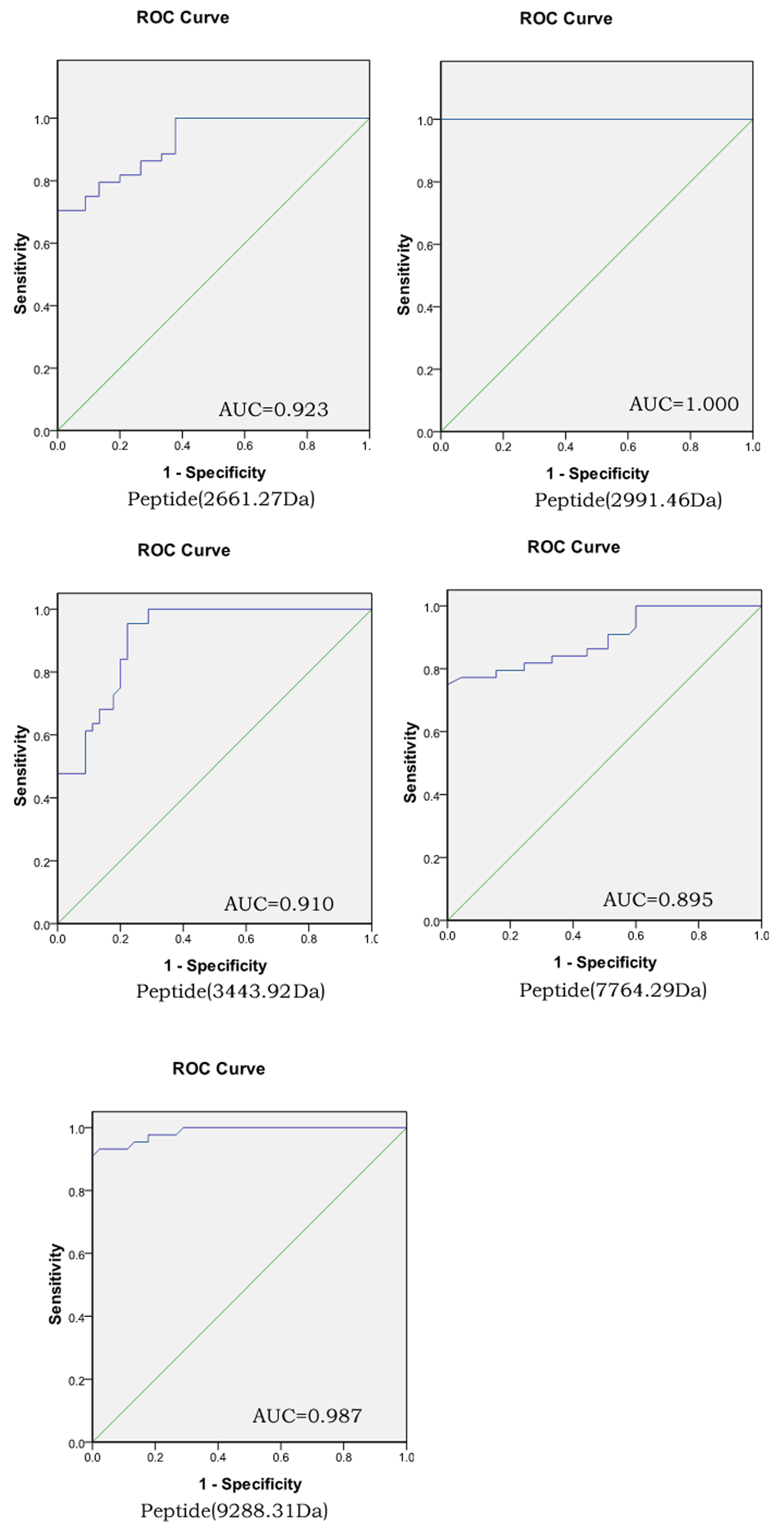

Supplement: Additional file 1: Figure S1. — ROC curves of the relative intensities of peptides differentiating patients with CR from RR. A. Area under the curve (AUC) of the peptide with MW of 2661.27 Da is 92.3%, representing higher diagnostic value for distinguishing CR from RR patients. B. AUC of the peptide with MW of 2991.46 Da is 100%, representing higher diagnostic value for distinguishing CR from RR patients. C. AUC of the peptide with MW of 3443.92 Da is 91%, representing higher diagnostic value for distinguishing CR from RR patients. D. AUC of the peptide with MW of 7764.29 Da is 89.5%, representing moderate diagnostic value for distinguishing CR from RR patients. E. AUC of the peptide with MW of 9288.31 Da is 98.7%, representing higher diagnostic value for distinguishing CR from RR patients. [file 12953_2014_49_MOESM1_ESM.jpeg]

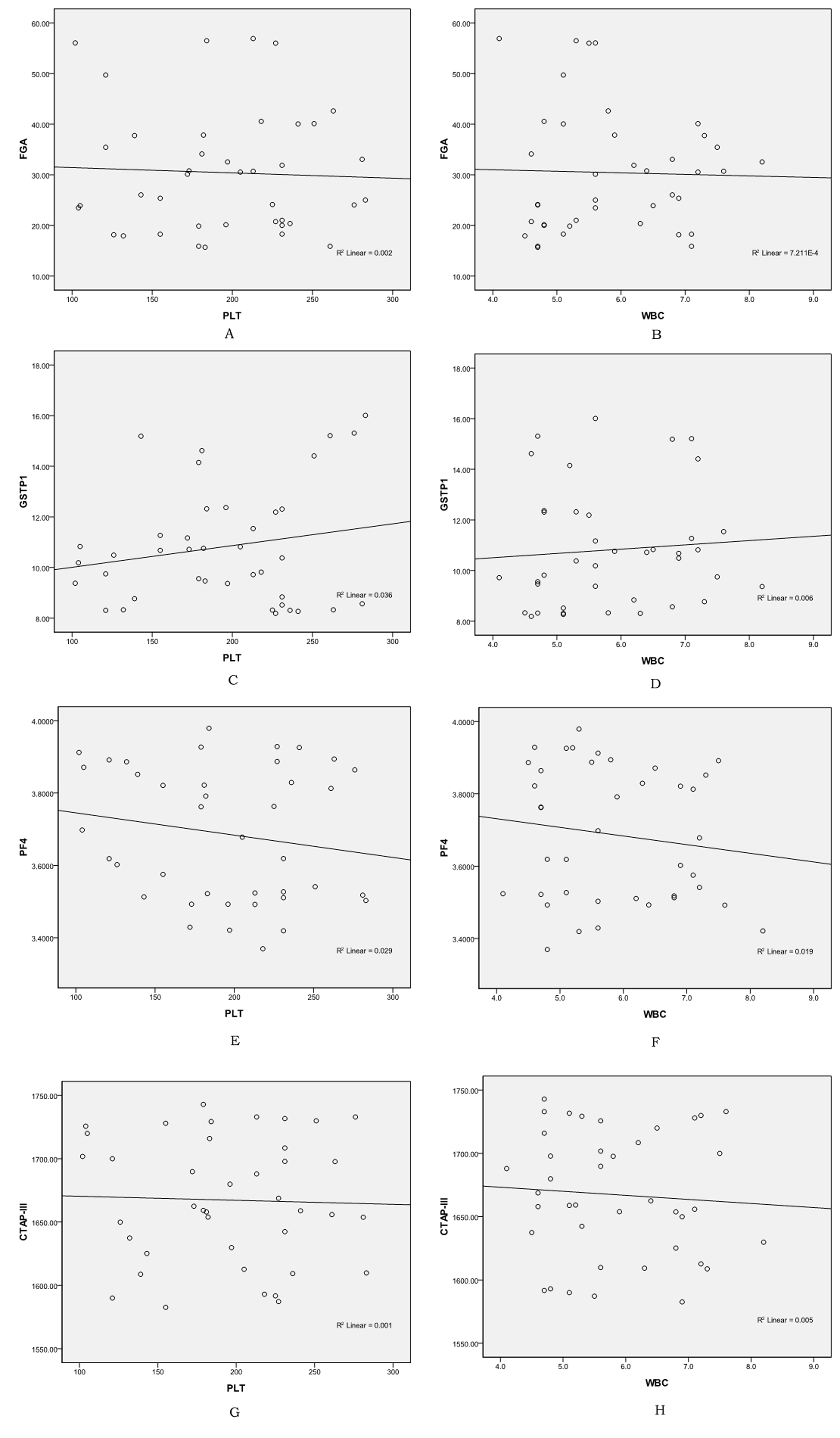

Supplement: Additional file 3: Figure S2. — Correlation analyses between contents of serum peptides and platelet/WBC counts in healthy control group. A. Correlation coefficient of FGA contents and platelet counts is 0.044 (p = 0.787). B. Correlation coefficient of FGA contents and WBC counts is 0.027 (p = 0.869). C. Correlation coefficient of GSTP1 contents and platelet counts is 0.191 (p = 0.238). D. Correlation coefficient of GSTP1 contents and WBC counts is 0.079 (p = 0.630). E. Correlation coefficient of PF4 contents and platelet counts is 0.171 (p = 0.292). F. Correlation coefficient of PF4 contents and WBC counts is 0.139 (p = 0.394). G. Correlation coefficient of CTAP-III contents and platelet counts is 0.033 (p = 0.838). H. Correlation coefficient of CTAP-III contents and WBC counts is 0.070 (p = 0.669). (FGA: fibrinogen alpha chain; GSTP1: glutathione S-transferase P1; PF4: platelet factor 4; CTAP-III: connective tissue active peptide III; WBC: white blood cell). [file 12953_2014_49_MOESM3_ESM.jpeg]

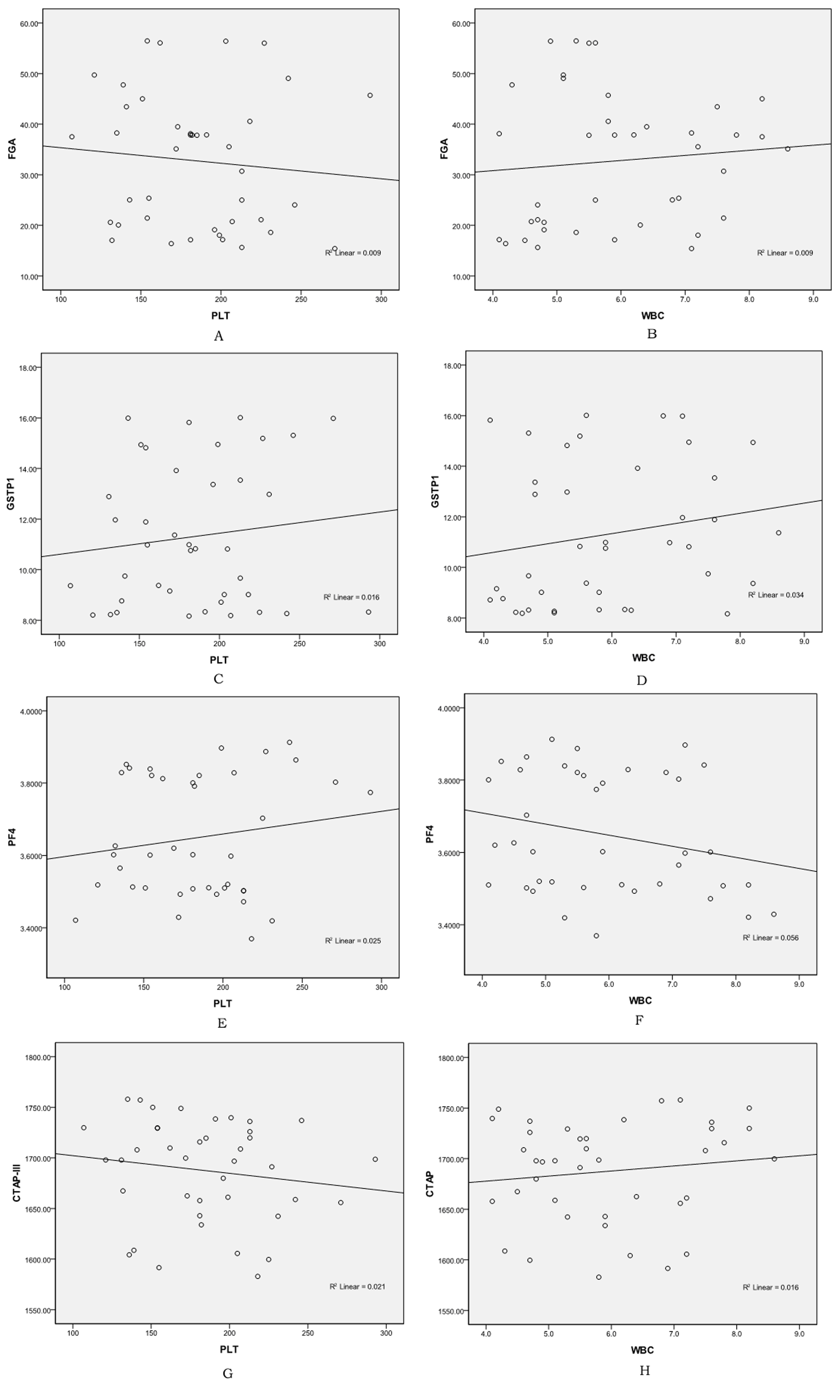

Supplement: Additional file 4: Figure S3. — Correlation analyses between contents of serum peptides and platelet/WBC counts in ALL complete remission group. A. Correlation coefficient of FGA contents and platelet counts is 0.097 (p = 0.552). B. Correlation coefficient of FGA contents and WBC counts is 0.097 (p = 0.552). C. Correlation coefficient of GSTP1 contents and platelet counts is 0.126 (p = 0.440). D. Correlation coefficient of GSTP1 contents and WBC counts is 0.185 (p = 0.254). E. Correlation coefficient of PF4 contents and platelet counts is 0.157 (p = 0.333). F. Correlation coefficient of PF4 contents and WBC counts is 0.236 (p = 0.143). G. Correlation coefficient of CTAP-III contents and platelet counts is 0.146 (p = 0.369). H. Correlation coefficient of CTAP-III contents and WBC counts is 0.128 (p = 0.431). (FGA: fibrinogen alpha chain; GSTP1: glutathione S-transferase P1; PF4: platelet factor 4; CTAP-III: connective tissue active peptide III; WBC: white blood cell). [file 12953_2014_49_MOESM4_ESM.jpeg]

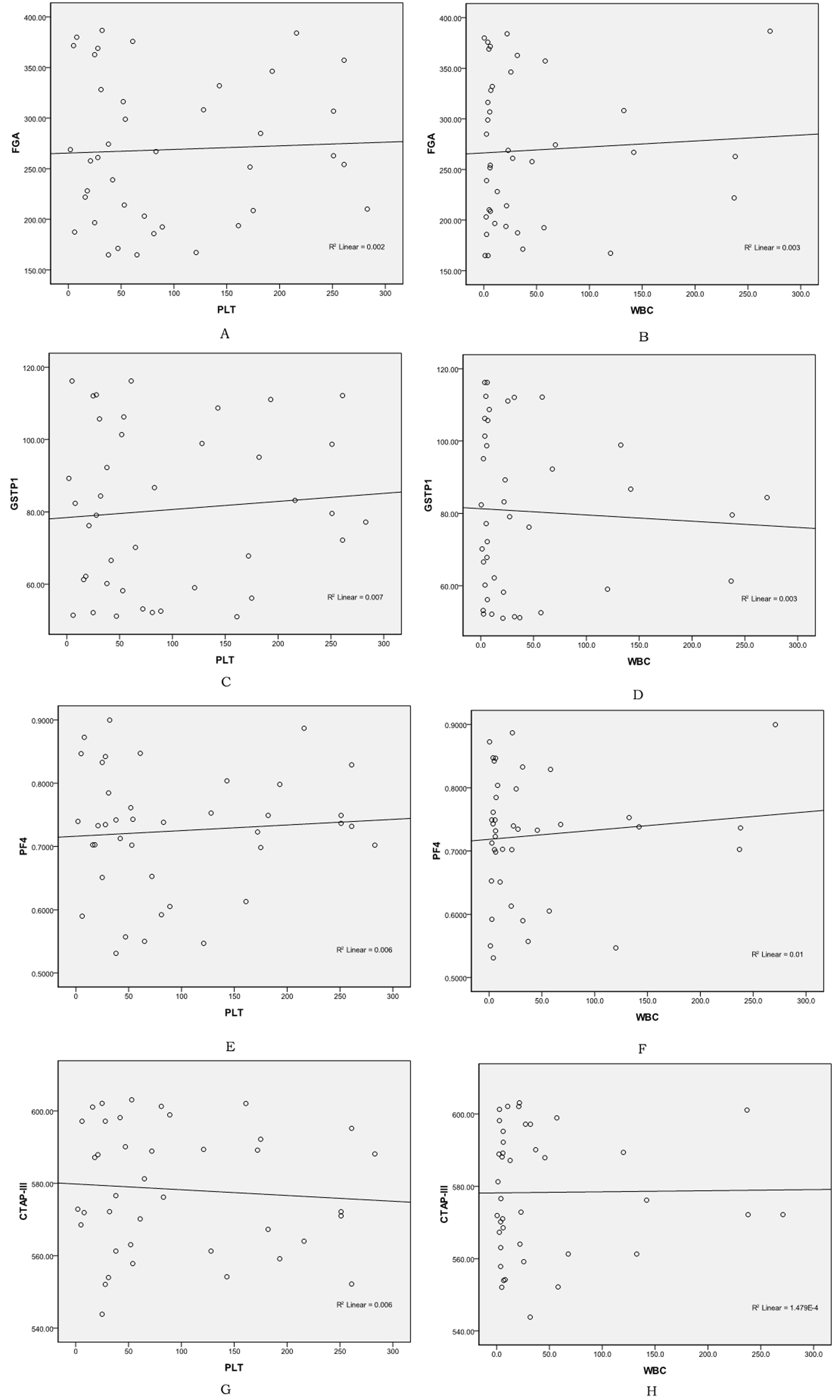

Supplement: Additional file 5: Figure S4. — Correlation analyses between contents of serum peptides and platelet/WBC counts in ALL refractory&relapsed group. A. Correlation coefficient of FGA contents and platelet counts is 0.043 (p = 0.792). B. Correlation coefficient of FGA contents and WBC counts is 0.057 (p = 0.728). C. Correlation coefficient of GSTP1 contents and platelet counts is 0.086 (p = 0.596). D. Correlation coefficient of GSTP1 contents and WBC counts is 0.054 (p = 0.741).E. Correlation coefficient of PF4 contents and platelet counts was 0.079 (p = 0.630). F. Correlation coefficient of PF4 contents and WBC counts is 0.102 (p = 0.529). G. Correlation coefficient of CTAP-III contents and platelet counts is 0.079 (p = 0.627). H. Correlation coefficient of CTAP-III contents and WBC counts is 0.012 (p = 0.941). (FGA: fibrinogen alpha chain; GSTP1: glutathione S-transferase P1; PF4: platelet factor 4; CTAP-III: connective tissue active peptide III; WBC: white blood cell). [file 12953_2014_49_MOESM5_ESM.jpeg]

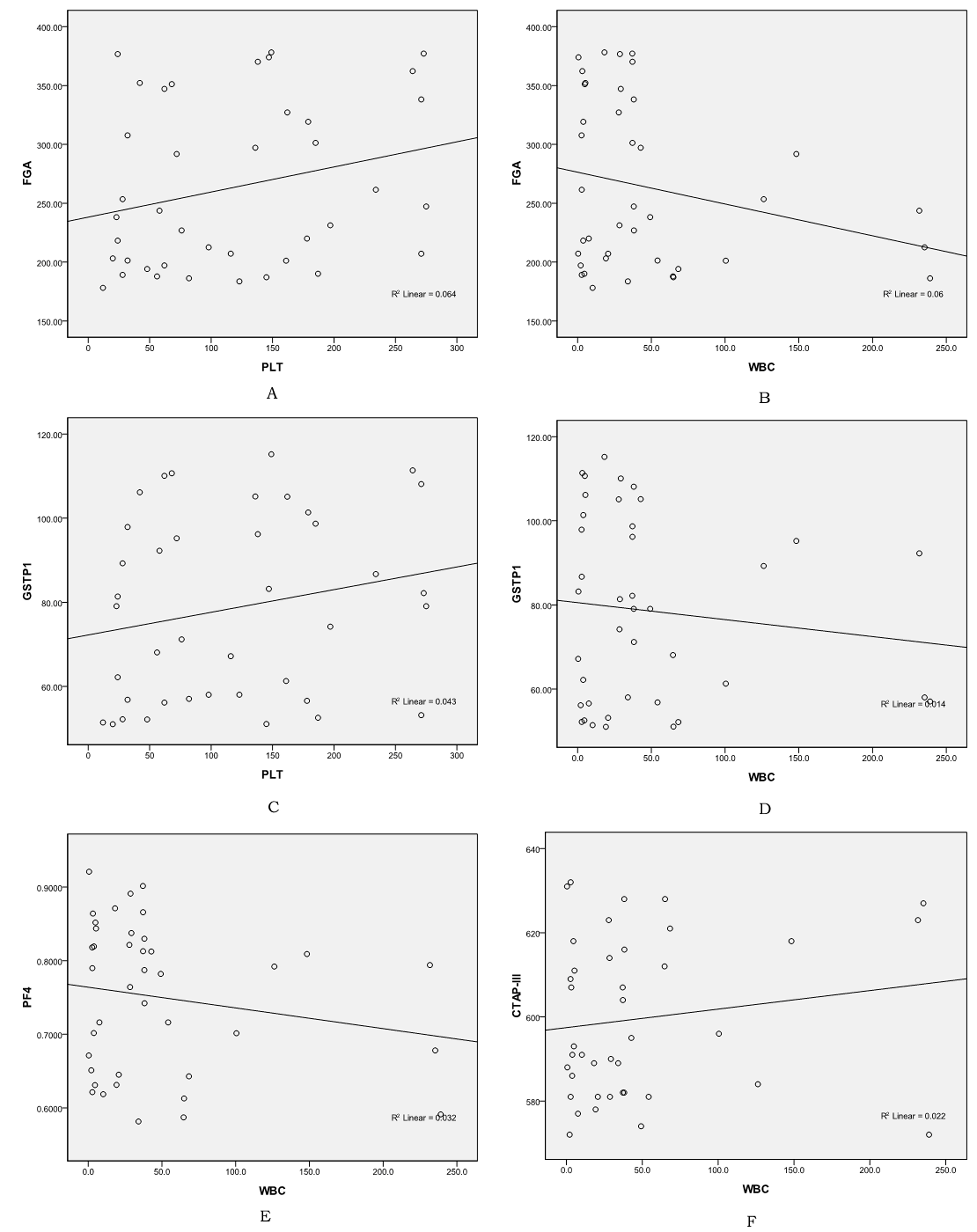

Supplement: Additional file 6: Figure S5. — Correlation analyses between contents of serum peptides and platelet/WBC counts in ALL newly diagnosed group. A. Correlation coefficient of FGA contents and platelet counts is 0.253 (p = 0.115). B. Correlation coefficient of FGA contents and WBC counts is 0.244 (p = 0.129). C. Correlation coefficient of GSTP1 contents and platelet counts is 0.207 (p = 0.200). D. Correlation coefficient of GSTP1 contents and WBC counts is 0.118 (p = 0.468). E. Correlation coefficient of PF4 contents and WBC counts is 0.178 (p = 0.271). F. Correlation coefficient of CTAP-III contents and WBC counts is 0.148 (p = 0.361). (FGA: fibrinogen alpha chain; GSTP1: glutathione S-transferase P1; PF4: platelet factor 4; CTAP-III: connective tissue active peptide III; WBC: white blood cell). [file 12953_2014_49_MOESM6_ESM.jpeg]

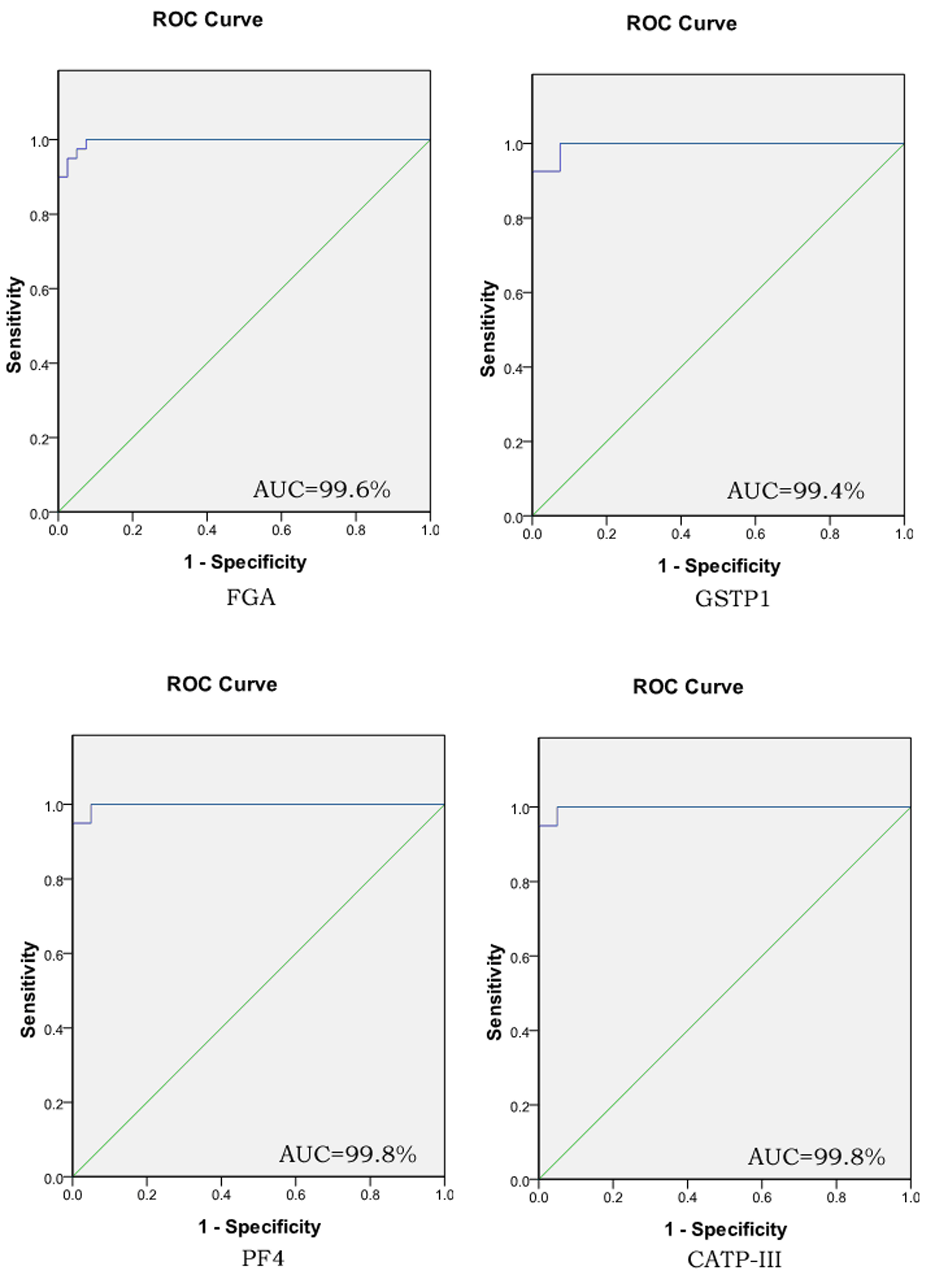

Supplement: Additional file 7: Figure S6. — ROC curves of the serum contents of proteins differentiating patients with CR from RR. A. Area under the curve (AUC) of FGA is 99.6%. B .AUC of GSTP1 is 99.4%. C. AUC of PF4 is 99.8%. D. AUC of CATP-III is 99.8%. AUCs of the four proteins are all ≥90%, representing higher diagnostic values of the serum contents of the four proteins for distinguishing CR from RR patients. (FGA: fibrinogen alpha chain; GSTP1: glutathione S-transferase P1; PF4: platelet factor 4; CTAP-III: connective tissue active peptide III). [file 12953_2014_49_MOESM7_ESM.jpeg]
