# Supplementary material for: Serum peptidome based biomarkers searching for monitoring minimal residual disease in adult acute lymphocytic leukemia
Source: Proteome Sci. 2014 Sep 16;12:49. doi: 10.1186/s12953-014-0049-y (PMC4195909; doi:10.1186/s12953-014-0049-y)

# Serum peptidome based biomarkers searching for monitoring minimal residual disease in adult acute lymphocytic leukemia

Ju Bai^1^, Aili He*^1^, Chen Huang^2^, Juan Yang^2^, Wanggang Zhang*^1^, Jianli Wang^1^, Yun Yang^1^, Pengyu Zhang^1^, Yang Zhang^1^, Fuling Zhou^1^

^1^ Department of Hematology, Second Affiliated Hospital, Medical School of Xi’an Jiaotong University, NO. 157 Xiwu Road, Xincheng District, Xi’an, Shaanxi, 710004, China

^2^ Department of Genetics and Molecular Biology, Medical school of Xi’an Jiaotong University/Key Laboratory of Environment and Disease-Related Gene, Ministry of Education, NO. 76 Western Yanta Road, Xi’an, Shaanxi, 710061, China

***Corresponding author:**

Aili He and Wanggang Zhang

Department of Hematology, Second Affiliated Hospital, Medical School of Xi’an Jiaotong University, NO. 157 Xiwu Road, Xincheng District, Xi’an, Shaanxi Province 710004, China; Phone: 086-29-87678325, Fax: 086-29-87678326, Email: [heaili@mail.xjtu.edu.cn](mailto:heaili@mail.xjtu.edu.cn); zhangwanggang2003@yahoo.com.

Email addresses:

**Ju Bai:**baiju.2008@stu.xjtu.edu.cn

**Aili He:**heaili@mail.xjtu.edu.cn

**Chen Huang:** hchen@mail.xjtu.edu.cn

**Juan Yang:** yangjuan0112@mail.xjtu.edu.cn

**Wanggang Zhang:**zhangwanggang2003@yahoo.com

**Jianli Wang:** wjianli@yahoo.com

**Yun Yang:** yangyun108@163.com

**Pengyu Zhang:** zhangpengyu2004@126.com

**Yang Zhang:** [zhangyang.1234567@163.com](mailto:zhangyang.1234567@163.com)

**Fuling Zhou:** [zhoufuling@163.com](mailto:zhoufuling@163.com)

# Abstract

## Background

The persistence of minimal residual disease (MRD) during therapy is the strongest adverse prognostic factor in acute lymphocytic leukemia (ALL). This study was to identify serum candidate peptides for monitoring MRD in adult ALL.

## Results

A total of 33 peptides in the molecular weight range of 1000-10000Da were detected using ClinProt system and statistically different between adult patients with ALL and healthy controls. Quick classifier (QC) algorithm was used to obtain a diagnostic model consisting of five peptides that could discriminate patients with ALL from controls with a high sensitivity (100%) and specificity (96.67%). The peptides in the QC model were identified as fibrinogen alpha chain (FGA), glutathione S-transferase P1 (GSTP1), isoform 1 of fibrinogen alpha chain precursor, platelet factor 4 (PF4) by high pressure/performance liquid chromatography mass spectrometry/mass spectrometry. Relative intensities of the five peptides were compared among ALL different groups for the potential importance of MRD evaluation in ALL. The peptides with increased relative intensities in newly diagnosed (ND) ALL patients were found to be decreased in their relative intensities after complete remission (CR) of adult ALL. When ALL patients were refractory&relapsed (RR), relative intensities of the peptides were elevated again. Peptides with decreased relative intensities in ND and RR ALL patients were found to be increased in their relative intensities when ALL patients achieved CR. The findings were validated by ELISA and western blot. Further linear regression analyses were performed to eliminate the influence of platelet and white blood cell counts on serum protein contents and indicated that there were no correlations between the contents of all four proteins (PF4, connective tissue active peptide III, FGA and GSTP1) and white blood cell or platelet counts in ALL different groups and healthy control.

## Conclusions

We speculate the five peptides, FGA, isoform 1 of fibrinogen alpha chain precursor, GSTP1, PF4 and connective tissue active peptide III would be potential biomarkers for forecasting relapse, monitoring MRD and evaluating therapeutic response in adult ALL.

**Key words:** serum peptidome, biomarker, adult acute lymphocytic leukemia, minimal residual disease

# Background

Acute lymphocytic leukemia (ALL) is a hematological malignancy with high heterogeneity. Adult ALL patients with different immunophenotypic, cytogenetic and molecular abnormalities manifest distinct prognostic and therapeutic implications . Considerable progress has been made in the therapeutics of ALL during the past two decades, however, the 5-year overall survival rates of adult ALL are within the 30-40% range, despite complete remission (CR) exceeding 90% in contemporary treatment series . The poor outcome of most adult ALL is due to an inevitable relapse after induction chemotherapy. Leukemia relapse is thought to result from minimal residual disease (MRD). MRD is the residual leukemia cells (as many as 10^8-9^) that remain following achievement of morphologic remission and are below the limits of detection using conventional microscopic and cytogenetic assessment of the bone marrow . MRD status best discriminated outcome after Phase 2 induction, when the relative risk of relapse was 8.95-fold higher in MRD-positive (≥10^-4^) patients and the 5-year relapse free survival was 15% compared to 71% in MRD-negative (<10^-4^) patients . Because MRD is an independent prognostic factor for relapse and survival of adult ALL, postremission MRD monitoring is now used to predict an impending relapse and to start preemptive salvage treatment in time .

Current methodologies to monitor MRD in ALL include flow cytometry (FCM) detection of aberrant immunophenotypes, which can detect 1 leukemic cell among 10000 normal cells (0.01%), and real-time polymerase chain reaction (RT-PCR) amplification of fusion transcripts, T-cell receptor (TCR) and immunoglobulin (Ig) genes, which has a sensitivity of 0.001% . However, all of the methods mentioned above have some limitations. First, a potential pitfall of FCM results from similarities between leukemic lymphoblasts and nonmalignant lymphoid precursors in various phases of regeneration or chemotherapy-induced alterations (phenotypic shifts) that may lead to false positivity. Moreover, FCM data interpretation requires a high level of expertise. Second, most adult ALL patients lack specific chromosome aberrations. Thus, RT-PCR amplification of fusion genes is currently limited to Philadelphia chromosome-positive (Ph+) ALL. Uncertain quantification, false-positivity resulting from cross-contamination, and false-negativity from RNA instability are caveats affecting fusion genes detecting. RT-PCR amplification of Ig and TCR genes are laborious and costly, because reagents for these types of assays are patient-specific. Furthermore, PCR analyses of Ig and TCR gene rearrangements need experienced personnel and standardization. In addition, oligoclonality and clonal evolution may produce false-negative results . Third, bone marrow cells are the specimens of FCM and RT-PCR based MRD monitoring. Bone marrow aspiration is invasive and increases the patients’ pain, whereas venepuncture is readily accepted by patients. Serum is easily accessible and contains a treasure trove of previously unstudied biomarkers that could reflect the ongoing physiologic or pathologic state of all tissues . Therefore, blood becomes one of the best sources for biomarkers researching.

The low-molecular-weight (LMW) region of the blood proteome, which is a mixture of small intact proteins plus fragments of the large proteins, is a treasure trove of diagnostic information ready to be harvested by nanotechnology . Serum peptidome refers to serum protein fragments and peptides whose molecular weights are less than 20kDa, particularly those of LMW . In recent years, mass spectrometry-based serum peptide profiling has been widely applied in the studies of markers in solid tumors . Preliminary findings showed that a group of serum peptides could be used for disease diagnosis and predicting disease progression.

Several serum peptidome researches on hematological malignancies had also been carried out. For instance, Albitar’s group explored the potential of peptidomic analysis of peripheral blood plasma, using ProteinChip-surface enhanced laser desorption ionization time of flight mass spectrometry (SELDI-TOF-MS) to predict recurrence of adult ALL, with correct predictions 84% to 92% . A classification model constructed by ProteinChip-SELDI-TOF-MS could discriminate the pediatric ALL samples from the controls with a sensitivity of 91.8% and a specificity of 90.0%. Furthermore, platelet factor (PF4), connective tissue activating peptide III (CTAP-III) and two fragments of C3a were identified by high pressure/performance liquid chromatography mass spectrometry/mass spectrometry (HPLC-MS/MS). They may be potential biomarkers to distinguish pediatric ALL patients from healthy controls (HCs) and pediatric acute myeloid leukemia (AML) patients . ProteinChip-SELDI-TOF-MS had the shortcoming of lower resolution and the peptides of interest could not be eluted for further identification . In recent years, magnetic beads with large separation capacity have replaced ProteinChip for sample preparation and enrichment. Because magnetic beads are high-throughput, simple and quick to operate, beads based-ClinProt technology has been widely used in the researches of serum peptidome in solid tumors . Previously, our group established a Quick classifier (QC) diagnostic model by ClinProt system, with a high sensitivity and specificity to discriminate adult AML patients from HCs. The three peptides were identified as ubiquitin-like modifier activating enzyme 1 (UBA1), isoform 1 of fibrinogen alpha chain precursor and PF4. Relative intensities of the three peptides were correlated with remission and clinical outcome of adult AML patients . Using the ClinProt system, a Supervised Neural Network Algorithm (SNN) diagnostic model was established for differentiating newly diagnosed (ND) multiple myeloma from HCs .

In our study, a comparative peptidomics method combining weak cation exchange beads (MB-WCX) and matrix assisted laser desorption ionization time of flight mass spectrometry (MALDI-TOF-MS) were used to analyze serum peptide profiles of adult ALL patients in ND group, CR group, refractory & relapsed (RR) group and HC group. We hypothesized that ClinProt-based serum peptidomics could detect differentially expressed peptides among different groups of adult ALL. Differences in peptides expression are reported in serum and bone marrow cells of ALL different groups and HC group. Moreover, differences in peptides expression are correlated with ALL therapeutic response and the time point of ALL relapse. These results suggest that the differentially expressed peptides would be appropriately adapted for predicting relapse, monitoring MRD and evaluating therapeutic response of adult ALL in clinical practice.

# Results

## Serum peptide fingerprints comparison between newly diagnosed ALL and healthy control

Despite varying peptide masses and spectrum intensities, the peak coefficient of variations (CVs) were all <14% in the within-run assays and <22% in the between-run assays. The CV value of the relative intensity of each peak in MALDI-TOF-MS was less than 30%, indicating that ClinProt system had good repeatability . To screen serum peptides of interest for adult ALL, comparative analyses of serum peptide fingerprints were performed between adult ALL patients and HCs by ClinProtools2.2 software. It was shown that peak number and intensity in serum peptide fingerprints of ND ALL patients were completely different from that of HCs (Figure 1). A total of 33 peptide peaks in the molecular weight range of 1000-10000Da were significantly different between the two groups (p<0.05). In ALL ND group, 13 peptides were up-regulated and 20 were down-regulated comparing with healthy controls (Table 1).

## Establishment of QC diagnostic model and blind verification

Genetic algorithm (GA), SNN and QC embedded in ClinProtools2.2 software were used to establish cross-validated classification model for distinguishing adult ALL from HCs. Among them, the QC model was composed of five peptides and had optimal distinction efficiency, in the training set with a sensitivity of 100% and a specificity of 96.67%.

In the QC model, comparing with healthy controls, peptides with molecular weight of 2661.27Da and 2991.46Da were upregulated in ALL ND group (Figure 2A, B), peptides with molecular weight of 3443.92Da, 7764.29Da and 9288.31Da were downregulated (Figure 2C, D, E). Blind verification showed that the QC model could correctly identify 52 cases out of total 54 ALL cases and 53 healthy cases from 55 HCs.

The diagnostic capability of each peak determined by the receiver operating characteristic (ROC) curve is reported in Figure 3.Areas under the curve (AUCs) of the peptides in the QC model were 92.56%, 89.22%, 91%, 86.56% and 90.22%, respectively. Generally, it is believed that the model has lower diagnostic value when AUC≤0.7, moderate diagnostic value when 0.7<AUC≤0.9, and higher diagnostic value when AUC>0.9. The AUCs of three peptide peaks in the QC diagnostic model were greater than 0.9, and the AUCs of the other two peaks were less than 0.9, but very close to 0.9.These results indicated that the QC diagnostic model composed of five peptides could distinguish the ND adult ALL from HCs.

## Peptides identification

The peptides in the QC model were purified and identified by HPLC-MS/MS. Data analysis software BioworksBrowser 3.3.1 SP1 was applied for Sequest™ searching. Positive protein identification was accepted for a peptide with Xcorr of greater than or equal to 3.50 for triply charged ions and 2.5 for doubly charged ions, and all with ΔCn≥0.1, peptide probability≤1e-3. Peptides with molecular weight of 2661.27Da, 2991.46Da, 3443.92Da and 7764.29Da were identified as peptide fragments of fibrinogen alpha chain, glutathione S-transferase P1, isoform 1 of fibrinogen alpha chain precursor 1 and platelet factor 4 (Table 2, Figure4, 5, 6, 7). However, peptide with molecular weight of 9288.31Da was not identified because of high molecular weight. Literature review suggested that it might be the connective tissue active peptide III .

## Relative intensities of the five peptides in QC model in ALL different groups

In order to explore the potential clinical significance of the five peptides (components of the QC diagnostic model), we compared the relative intensities of the five peptides among ALL ND group, CR group, RR group and HC group (detail data were showed in Table 3). Kruskal-Wallis rank sum test was performed to ascertain the significant level of difference among the groups.

Compared with HC group and CR group, the relative intensities of peptides with molecular weight of 2661.27Da and 2991.46Da were significantly elevated in ALL ND group (p<0.001, p<0.001) and RR group (p<0.001, p<0.001). No significant differences were observed in the relative intensities of peptides with molecular weight of 2661.27Da and 2991.46Da between the ND group and the RR group (p=0.76, p=0.59) nor the HC group and the CR group (p=0.67, p=0.38).

A significant decrease in relative intensities of peptides with molecular weight of 3443.92Da, 7764.29Da and 9288.31Da were found in ND group and RR group, compared with HC group (p<0.001, p<0.001, p<0.001) and CR group (p<0.001, p<0.001, p<0.001). No significant differences were observed in the relative intensities of peptides with molecular weight of 3443.92Da, 7764.29Da and 9288.31Da between the ND group and the RR group (p=0.62, p=0.75, p=0.26) nor the HC group and the CR group (p=0.71, p=0.23, p=0.67).

To define the threshold for each peptide that could be used to discriminate CR and RR patients, ROC analysis was performed. The cutoff values of the relative intensities of the five peptides were 8.06, 4.87, 1.75, 2.02 and 1.42, respectively (Suppl. Figure 1).

In addition, we tested whether the relative intensities of the five peptides were correlated with subtypes, gender or age in ND ALL patients. The results showed that the relative intensities of the five peptides were not different between B-ALL and T-ALL (p>0.05). It is important to point out that no statistical differences were observed in relative intensities of the five peptides when ND ALL patients were classified into two groups according to gender or age (p>0.05, p>0.05) (Suppl. Table).

## Impact of the relative intensities of the five peptides on relapse

During the follow-up, 39 newly diagnosed ALL patients underwent relapse ultimately. The median relapse time was 16 months (range 5-29 months) after initial diagnosis. Our analyses included patients who relapsed very early (during first 18 months after initial diagnosis: 56.4%, 22/39) and early (between 18 and 30 months: 43.6%, 17/39), whereas late relapse patients (>30 months) were absent. Correlation plots between the peak intensities of the five peptides and the relapse time (months) were drawn. The relative intensities of peptides with molecular weight of 2661.27Da and 3443.92Da were not correlated with time point of ALL relapse. Correlation coefficients were 0.115 (p=0.347) and 0.071 (p=0.666) (Figure 8A, C). The relative intensities of peptides with molecular weight of 2991.46Da, 7764.29Da and 9288.31Da were correlated with time point of ALL relapse. Correlation coefficients were 0.959 (p<0.01), 0.916 (p<0.01) and 0.921 (p<0.01) (Figure 8B, D, E).

## Validation of protein fragments by immunoblotting

The levels of FGA, GSTP1 and PF4 protein in ALL and control samples were determined by western blot analysis. The levels of FGA and GSTP1 protein differed among the leukemia cells in distinct ALL groups and normal cells by immunoblotting (Figure 9A). Quantification of bands from western blot analysis showed significantly increased levels of FGA and GSTP1 protein in ND and RR ALL samples, when compared with age-matched control and CR ALL samples (p=0.0049, p=0.0012, Figure 9B, C). Weak PF4 immunoreactive bands were seen in ND and RR ALL cases (Figure 8A). Relative to age-matched CR ALL and healthy control samples, quantification of bands from western blot analysis showed significantly decreased levels of PF4 in ND and RR ALL samples (p = 0.0061, Figure 9D).

## Determination of serum proteins by ELISA

Serum FGA, GSTP1, PF4 and CTAP-III contents were detected by enzyme linked immunosorbent assay (ELISA) in 40 ALL-ND, 40 ALL-CR, 40 ALL-RR patients and 40 HCs. FGA and GSTP1 contents in ALL-ND group (263.48±70.19µg/L, 78.62± 21.73µg/L) and ALL-RR group (268.92±71.51µg/L, 80.57±22.15 µg/L) were significantly increased (p=2.8641E-7, p=3.1879E-5; p=1.9692E-7, p=3.3821E-5), compared with HC group (30.44±12.15µg/L,10.82±2.31µg/L) and CR group (32.76± 13.28µg/L,11.31 ±2.79µg/L). Differences of the platelet and white blood cell (WBC) counts are evident between diseased and disease free blood samples. Correlation analyses revealed that no correlation between FGA, GSTP1 contents and platelet counts or WBC counts in all four groups (HC, CR, RR and ND) (Suppl. Figure 2A, B,C,D; Suppl. Figure 3A,B,C,D; Suppl. Figure 4A,B,C,D; Suppl. Figure 5A,B,C,D).

The PF4 and CTAP-III contents were found to be decreased in patients with ND ALL (0.7506±0.1003µg/L, 599.55±18.86µg/L) and RR ALL (0.7246±0.0972µg/L, 578.31 ±17.24µg/L) in comparison with HCs (3.6872±0.1853µg/L, 1667.33± 49.36µg/L) and CR ALL patients (3.6496±0.1669µg/L, 1687.48±50.19µg/L) (p=1.4595E-5, p=3.2170 E-5; p=4.5269E-6, p=3.7912 E-6). PF4 and CTAP-III are platelet-derived chemokines. Correlation analyses were further done between platelet counts and PF4 contents as well as CTAP-III contents in ND ALL patients to eliminate the influence of platelet counts on serum PF4 and CTAP-III contents. Correlation coefficients were 0.251 (p=0.119) and 0.078 (p=0.632), and no correlations were found between them (Figure 10). The contents of the two proteins had no correlation with platelet or WBC counts in other groups (HC,CR and RR) ( Suppl. Figure 2E,F,G,H; Suppl. Figure 3 E,F,G, H; Suppl. Figure 4 E,F,G,H; Suppl. Figure 5E,F).

ROC analyses were performed to define thresholds for each peptide that could be used to discriminate CR and RR patients. ROC analyses yielded cutoff values of the serum contents of FGA (110.72µg/L), GSTP1 (33.51µg/L), PF4 (2.1347µg/L), CTAP-III (1093.04µg/L), respectively (Suppl. Figure 6).

# Discussion

Published data on MRD assessment in adult ALL have shown a strong correlation between MRD response and outcome as well as the prognostic value of MRD reappearance for hematologic relapse. By using ClinProt-based serum peptidomics, we were able to identify five differentially expressed peptides (components of the QC model) in the serum of adult ALL patients in ND group, CR group, refractory & relapsed (RR) group and HC group. The candidate peptides were identified as FGA, GSTP1, isoform 1 of fibrinogen alpha chain precursor, PF4 and CTAP-III by HPLC-MS/MS and validated by western blot and ELISA. They were correlated with ALL therapeutic response and the time point of ALL relapse. Thus, they would be appropriately adapted for predicting relapse, monitoring MRD and predicting therapeutic response of adult ALL in clinical practice.

Our QC diagnostic model composed of five peptides had optimal distinction efficiency between ND ALL patients and HCs. In the training set, QC model showed a high sensitivity (100%) and specificity (96.67%). 52/54 ALL cases were correctly identified for blind validation. The diagnostic capabilities of the five peptides, determined by the ROC curve, were proved to be highly and moderately accurate (AUC > 0.90 or close to 0.9). The data suggest that the five peptides may be diagnostic biomarkers for adult ALL. The specimen volumes require augmentation for further validation of their clinical diagnostic/prediction value. Moreover, functional studies of the five peptides are necessary and conducive to ALL pathogenesis clarification.

In recent years, mass spectrometry-based proteomics technology has been applied in some researches on hematologic malignancies, mainly including the traditional two-dimensional gel electrophoresis (2-DE) based separation technology combining with MALDI-TOF-MS or Proteinchip-SELDI-TOF-MS technology. Our study utilized MB-WCX technology for serum LWM peptides purification, MALDI-TOF-MS technology for mass spectra acquisition, and highly sophisticated data mining algorithms for inspection and comparison of data sets as well as for the discovery of complex biomarker pattern models. Traditional 2-DE suffers from low degree of automation, time-consuming and poor repeatability. The target surface of protein chip has low-capacity binding sites, which influences the detection of spectra of proteins and peptides. MALDI-TOF MS provides optimal performance for m/z from 1 to 15 kDa. As the m/z increases (>15 kDa), resolution and mass accuracy progressively decrease. In the low m/z range(<1 kDa), there is a higher background from ionized matrix molecules. Comparing with the other two types of beads, MB-WCX beads group provided the best proteomic pattern, the most average peak numbers, the highest peak intensities, and the best capturing ability of low abundance proteins or peptides in serum samples.

Our study showed that the relative intensities of the five peptides were correlated with response of ALL. Results were validated by western blot and ELISA. Differences of the platelet and WBC counts are evident between diseased and disease free blood samples. ELISA results indicated that contents of the five peptides had no correlation with WBC or PLT counts in ALL different groups and HC group. Although B- and T-lineage ALL differ significantly in their immunophenotypic, genetic and molecular markers, no significant differences were found between B-ALL and T-ALL in the relative intensities of the five peptides in our study, representing that the peptides were pan-leukemic biomarkers. These results suggest that the peptides may be potential markers for predicting treatment response and monitoring MRD in adult ALL.

Correlation analyses showed that relative intensities of peptides with molecular weight of 2991.46Da, 7764.29Da and 9288.31Da were correlated with time point of ALL relapse. Adult newly diagnosed ALL patients with very early relapse presented higher relative intensity of peptide with molecular weight of 2991.46Da. Relative intensities of peptides with molecular weight of 7764.29Da and 9288.31Da were lower in newly diagnosed adult ALL patients who relapsed very early. Considering the strong positive correlations found between peptide levels and time of relapse, these peptides had the potential for disease monitoring and early relapse diagnosis.

A variety of biomolecules are involved in the development and relapse of ALL. Previously, translocation t(9;22) (q34;q11) (Ph chromosome) with the BCR-ABL1 fusion was found in 30% adult ALL . It is conducive to imatinib targeted therapy, predicting prognosis and monitoring MRD for ALL. Our study showed the serum peptidomic markers for predicting treatment response and monitoring MRD in adult ALL. Moreover, recent papers reported that several metabolic biomarkers of ALL from bone marrow/plasma samples may represent an underlying metabolic pathway associated with disease progression and relapse or remission, such as glycerophospholipid metabolism , GPEtn, GPCho ,amino acid metabolites and derivatives . The development and relapse of ALL are multi-step and multi-stage processes. Therefore, combining proteomic data with genomic and metabolomic data would provide a much better understanding of leukemogenesis and relapse of ALL.

Pathogenesis and progression of ALL, as well as response to therapies, involve a series of genetic and epigenetic events. Versatile changes of the peptide levels in patients’ serums may be reflection of these genetic/epigenetic changes during disease evolvements. And potential functions of these proteins/peptides in ALL evolvements are waiting to be elucidated. Fibrinogen circulates in plasma as a dimer, composed of three pairs of unequal polypeptide chains denoted alpha chain, beta chain and gamma chain. Extensive researches indicated that fibrinogen was overexpressed in many malignant tumors and could be an independent prognostic parameter for the disease-free, distant-free and overall survival for patients with malignancies, such as pancreatic cancer , renal cell carcinoma , endometrial carcinoma, cervical cancer, oesophageal squamous cell carcinoma and so on. Fibrinogen was considered as an acute phase reactant protein and raised in tumour progression and increased synthesis of fibrinogen was associated with an ongoing inflammatory response to tumor. Some studies suggested that tumor cells could promote coagulation process by interacting with endothelial cells and platelets, then by releasing active biological substances that activate platelets, which lead to high level of fibrinogen in the cancer blood. The relative intensity of FGA was increased in ND ALL and decreased when patients received CR. When ALL patients underwent recurrence, relative intensity of FGA was elevated again. The results were validated by ELISA and western blot. Relative intensities of isoform 1 of fibrinogen alpha chain precursor peptide fragment were opposite to that of FGA in ALL different groups. It appears that a large part of the human serum peptidome detected by MALDI-TOF-MS is produced ex vivo by degradation of endogenous substrates through endogenous proteases . The peptide (2661.27Da) identified (D605-V629) is localized close to the C-terminal end of fibrinogen alpha chain preproprotein and has a proteolytic cleavage site with a high susceptibility to plasmin attack. However, there is no proteolytic cleavage site in the identified peptide (3443.92Da, H511-V541). Therefore, the peptide (2661.27Da) could be a putative plasmin-generated proteolytic fragment and the overexpression of FGA peptide fragment in ND-ALL was due to a hypothetical increased enzymatic activity able to attack the C-terminal end of FGA.

Glutathione S-transferase P1 (GSTP1), the main drug metabolism and stress response signalling protein, can protect cells from cytotoxic and carcinogenic agents. It has been found to be correlated with multidrug resistance and poor clinical prognosis of tumors. GSTP1 was upregulated in a variety of tumors . Apart from aberrant protein expression levels, attention has been directed toward the association of GSTP1 polymorphisms with the risk of cancers . GSTP1 was found to be involved in the drug resistance, and inhibition of GSTP1 expression could reverse the drug resistance and induce tumour cell apoptosis . The study of GSTP1 genetic polymorphisms in pediatric ALL showed that GSTP1 genotype was unrelated to genetic susceptibility of ALL, but GSTP1*V105 was involved in ALL relapse. Studies suggested that the GSTP1Val/Val genotype might be considered as risk genotype for developing ALL and was correlated with poor prognosis. Our research showed that a positive correlation existed between GSTP1 level and ALL treatment response and newly diagnosed ALL patients with higher relative intensity of GSTP1 peptide were prone to an earlier relapse. It is speculated that GSTP1 may play an important role in ALL progression and chemotherapy resistance. The specific mechanism and genetic polymorphism of GSTP1 in adult ALL need further study.

PF4 and CTAP-III are two platelet-associated chemokines that modulate tumor angiogenesis, inflammation within the tumor microenvironment, and tumor growth. Vermeulen found that average levels of PF4 and CTAP-III were down-regulated in the serum of benzene-exposed workers in comparison with control subjects . Several proteomic studies revealed that PF4 and CTAP-III were decreased in acute leukemia and MDS comparing with HCs . Our study showed that relative intensities of PF4 and CTAP-III were negatively correlated with adult ALL response. Moreover, newly diagnosed ALL patients with lower relative intensities of PF4 and CTAP-III were prone to an earlier relapse. By immunoblotting, PF4 protein was significantly decreased in ND and RR ALL cells. The serum PF4 content was correlated with treatment response. These findings are consistent with Kim’s study . PF4 and CTAP-III are platelet-derived chemokines, whereas there were always reduced platelets and WBC counts in hematologic malignancies. Previous studies revealed that lower PF4 expression was not due to thrombocytopenia. Our ELISA results demonstrated that no differences were found in serum PF4 and CTAP-III contents between ALL with and without reduced platelets/WBC counts. Linear regression analyses showed no correlation between PF4/CTAP-III contents and platelet or WBC counts. It follows that the reduction of serum PF4 and CTAP-III contents in ND ALL are not due to thrombocytopenia and hypoleucocytosis. PF4 is a major antiangiogenic factor. We found that PF4 was downregulated in ND and RR ALL, which may indicate that the antiangiogenic activity of PF4 was compromised in these patients. Loss of anti-angiogenesis activity and the resultant imbalance between pro- and anti-angiogenesis may contribute to increased proliferation and infiltration of ALL cells. This is probably one of the reasons why PF4 expression is negatively associated with clinical outcome of ALL.

# Conclusions

In conclusion, the ALL QC model constructed by application of ClinProt system had a high sensitivity and specificity to discriminate between ALL patients and healthy controls. The relative intensities of the peptides in the QC model were correlated with ALL treatment response and time point of relapse. These findings were validated by ELISA and western-blot. We speculate these peptides, such as fibrinogen alpha chain, isoform 1 of fibrinogen alpha chain precursor and glutathione S-transferase P1, platelet factor 4, connective tissue active peptide III can be used as potential markers for predicting relapse, assessing treatment response and monitoring minimal residual disease in adult ALL.

# Methods

## Study population

This study was approved by Ethics Committee of the Second Affiliated Hospital of Medical School of Xi’an Jiaotong University. 84 serum samples were collected from adult ND ALL patients who had been confirmed by lymphocytic cytological diagnosis based on the FAB classification system in the Second Affiliated Hospital of Xi’an Jiaotong University during a given period of time (2009.12–2012.8). At the same time, 84 healthy control serum samples were collected from adults who took health examinations and had not been diagnosed with any disease. In addition, 44 serum samples from ALL patients with hematologic CR and 45 serum samples from RR ALL patients were obtained (details in Table 4). 45 RR ALL patients consisted of 39 early relapse patients and 6 refractory patients. The response criteria of ALL were set according to the definition of response in adult ALL . The CR and RR serum samples in our study were taken at d28 from initiation of post-induction therapy. The ND adult ALL patients were treated with VDP (Vinorelbine 25-30mg/m^2^, iv, d1, 8, 15, 22; Pirarubicin 20-25mg/m^2^, iv, d1, 2, 3; Prednisone 60mg/m^2^, po, d1-28), VDLP (Vinorelbine 25-30mg/m^2^, iv, d1, 8, 15, 22; Pirarubicin 20-25mg/m^2^, iv, d1, 8, 15, 22; L-Asparaginase 10000U, iv, d17-28; Prednisone 60mg/m^2^, po, d1-28). Ph (+) ALL patients also received oral imatinib (400-600mg/d). All donors obtained informed consents.

## Serum peptides purification

Serum samples were collected according to standard protocol. Fasting blood samples were collected from patients in the morning and allowed to clot at room temperature for 2 h. Sera were then separated by centrifugation at 2500 rpm for 10min and stored at -80℃ until analysis. The length of cryopreservation period for each serum sample was less than 6 months. For the reproducibility experiments, serum from each ALL patient and each HC were processed using the same MALDI-TOF-MS instrument to run three within-run assays and three between-run assays.

MB-WCX beads kit was purchased from Bruker Daltonics Inc. (Billerica, MA·USA) and used to extract serum peptides. All purifications were performed in a one-step procedure according to manufacturers’ instructions through a standard protocol (ClinProt^TM^, Bruker Daltonics). Detail operation steps were done as previously described . Briefly, first, 10 µL beads, 10 µL binding solution (BS) and 5 µL serum samples were mixed by pipetting in a 200μL Orcugen sample tube and incubated for 5 min. The tube was then placed on a magnetic bead separation device for 1 min to collect the beads on the tube wall. The supernatant was removed and 100 µL MB washing solution (WS) was added and mixed thoroughly with the beads. After washing three times, the supernatant was removed and 5 µL MB eluting solution (ES) was added. The beads were collected on the tube wall in the separation device for 2 min. Finally, the clarified supernatant was transferred to a fresh tube with 5μl stabilization buffer (SB) and stored at −20 °C.

## Data acquisition by MALDI-TOF-MS

1) 1μL eluted sample was spotted onto a MALDI-TOF AnchorChip^TM^ target (600μm anchor diameter) and air-drying at room temperature, then 1μL matrix (0.3mg/ml HCCA, 50%ACN, 2%TFA) was spotted onto MALDI-TOF AnchorChip target. 2) AnchorChip target was placed into the Microflex mass spectrometer (Bruker Daltonics). 3) Samples were detected after calibration of the instrument by ClinProt standard, scan range was 1-l0KD. FlexControl2.2 software was applied to data acquisition and peptide profilings were gained constituted by different mass to charge ratios (m/z).

## Data processing and statistical analysis

Original mass spectra were normalized by Flexanalysis3.0 software, including smoothing and substrate baseline. Thereafter, we selected statistical algorithm built-in Clinprotools2.2 software for statistical analyses and acquired different expressed peptides. Wilcoxon rank sum test was performed to compare relative intensities differences of peptide peaks between newly diagnosed ALL and healthy controls. Statistical significance was defined as p<0.05. Relative intensity differences of the peptide peaks in each group were analysed by Kruskal-Wallis rank sum test. For multiple comparisons among the four groups, significant level was adjusted to 0.0083(0.05/4(4-1)/2). Statistical significance was defined as p<0.0083. GA, SNN and QC algorithm were applied to establish diagnostic model for distinguishing ALL from HCs. Correlation analysis was used for linear regression analysis. ROC analyses were performed to calculate AUCs to define threshold for each peptide that could be used to discriminate CR and RR patients.

## Identification of peptide biomarkers

Nano-LC/ESI-MS/MS system consisting of an Aquity UPLC system (Waters Corporation, Milford, USA) and a LTQ Obitrap XL mass spectrometer (Thermo Fisher, Waltham, MA, USA) equipped with a nano-ESI source (Michrom Bioresources, Auburn, USA) was utilized to perform peptide sequencing and identification. The peptide solutions purified by magnetic beads, were loaded to a C18 trap column (nanoACQUITY) (180 μm×20mm×5μm (symmetry)). The flow rate was 15μl/min. Then the desalted peptides were analyzed by C18 analytical column (nanoACQUITY) (75 μm×150 mm×3.5μm (symmetry)) at a flow rate of 400nl/min for 60 min. The mobile phases A (5%acetonitrile, 0.1%formic acid) and B (95%acetonitrile, 0.1% formic acid) were used for analytical columns. The gradient elution profile was as follows: 5%B–45%B–80%B –80%B–5%B–5%B in 60 min. The spray voltage was 1.8kV. MS scan time was 60min. Experimental mode were data dependent and dynamic exclusion, scilicet MS/MS spectra were limited to two consecutive scans per precursor ion within 10 s followed by 90 s of dynamic exclusion. Mass scan range was from m/z 400 to 2000. MS scan used Obitrap, resolution was set at 100000. CID and MS/MS scan employed LTQ. In MS spectrogram, we selected single isotope composed of 10 ions with strongest intensity as parent ion for MS/MS. Single charge was excluded and not as parent ion. We applied data analysis software Bioworks Browser 3.3.1 SP1 for Sequest™ retrieving. Retrieval Database was International Protein Index (IPI human v3.45 fasta with 71983 entries). Parent ion error and fragment ions error were set at 100ppm and 1Da, respectively. Digested mode was non-digested and variable modification was methionine oxidation.

## Western blot analysis for validation

Sodium dodecyl sulfate polyacrylamide gel electrophoresis (SDS-PAGE) and immunoblotting were performed essentially as described elsewhere . Briefly, cell pellets were resuspended on ice in lysis buffer containing 10mM Tris-HCl (pH 7.4), 5mM MgCl_2_, 1% Triton X-100, 100mM NaCl, 10mM NaF, 1mM Na_3_VO_4_ and a protease inhibitor cocktail. After sonication, cellular proteins were separated on an SDS-polyacrylamide gel and transferred to polyvinylidene fluoride membranes (Roche Diagnostics Corporation, Indianapolis, Indiana United States), which were probed with the appropriate primary antibodies. Immunoreactivity was detected with the relevant horseradish peroxidase-labeled secondary antibodies which, in turn, were visualized on an Image Reader Tano-5500 (Tano, Shanghai, China) using chemiluminescence substrate reagent purchased from 7 sea pharmtech (Shanghai, China). For quantification of the data, the images were further analyzed on the same instrument using 2D Densitometry Image Analyzer IPP 7.0 software (Tano, Shanghai, China).

## ELISA determination of serum proteins

Serum contents of FGA, GSTP1, PF4 and CTAP-III were assayed by ELISA and compared in 40 ND, 40 CR, 40 RR ALL patients and 40 HCs. Detailed procedures were according to manufacturers’ instructions of ELISA kit (R&D, USA). Furthermore, we analyzed the correlation of serum protein contents and platelet as well as WBC counts in different ALL patients and HCs. SNK-q test was done through the statistical software SPSS17.0. For multiple comparisons among the four groups, significant level was adjusted to 0.0083(0.05/4(4-1)/2). Correlation analysis used linear regression analysis. Statistical significance was defined as p < 0.05.

# Abbreviations

ALL: acute lymphocytic leukemia; MRD: minimal residual disease; CR: complete remission; FCM: flow cytometry; RT-PCR: real-time polymerase chain reaction; TCR: T-cell receptor; Ig: immunoglobulin; LMW: low-molecular-weight; SELDI-TOF-MS: surface enhanced laser desorption ionization time of flight mass spectrometry; PF4: platelet factor; CTAP-III: connective tissue activating peptide III; HPLC-MS/MS: high performance liquid chromatography tandem mass spectrometry/mass spectrometry; HCs: healthy controls; AML: acute myeloid leukemia; QC: quick classifier; UBA1:ubiquitin-like modifier activating enzyme 1; RI: relative intensity; SNN: supervised neural network; ND: newly diagnosed; MB-WCX: weak cation exchange magnetic beads; MALDI-TOF-MS: matrix assisted laser desorption ionization time of flight mass spectrometry; RR: refractory&relapsed; GSTP1: glutathione S-transferase P1; FGA: fibrinogen alpha chain; CV: coefficient of variation; GA: genetic algorithm; AUC: area under the curve; ROC: receiver operating characteristic;2-DE:two-dimensional gel electrophoresis; ELISA: enzyme linked immunosorbent assay; BS: binding solution; WS: washing solution; ES: eluting solution; SB: stabilization buffer; SDS-PAGE: sodium dodecyl sulfate polyacrylamide gel electrophoresis.

# Competing interests

The authors declare no conflict of possible interests. We have no conflicts of interest with the trademarks.

# Authors' contributions

JB and YZ were involved in serum peptide purification and writing the draft of the manuscript; JY performed serum peptide profiling data acquisition and processing; YY and JLW were involved in serum samples and clinical data collection; PYZ and FLZ performed ELISA and western-blot validation; ALH, WGZ and CH designed the experiments and supervised the research manuscript. All authors read and approved the manuscript.

# Acknowledgements

This work was supported by Program for Changjiang Scholars and Innovative Research Team in University (PCSIRT:1171), the National Natural Science Foundation of China (NSFC) (NO: 81100374), Science and Technology Project of Xi'an (Social development guide plan,SF1317(1)).

# References

1. Faderl S, O'Brien S, Pui CH, Stock W, Wetzler M, Hoelzer D, Kantarjian HM: **Adult acute lymphoblastic leukemia: concepts and strategies.** *Cancer* 2010, **116:** 1165-1176.

2. Ribera JM: **Advances in acute lymphoblastic leukemia in adults.** *Curr Opin Oncol* 2011, **23:** 692-699.

3. Ribera JM: **Acute lymphoblastic leukemia in adults.** *Pediatr Rep* 2011, **3 Suppl 2:** e1.

4. Bruggemann M, Raff T, Kneba M: **Has MRD monitoring superseded other prognostic factors in adult ALL?** *BLOOD* 2012, **120:** 4470-4481.

5. Bruggemann M, Raff T, Flohr T, Gokbuget N, Nakao M, Droese J, Luschen S, Pott C, Ritgen M, Scheuring U, Horst HA, Thiel E, Hoelzer D, Bartram CR, Kneba M: **Clinical significance of minimal residual disease quantification in adult patients with standard-risk acute lymphoblastic leukemia.** *BLOOD* 2006, **107:** 1116-1123.

6. Bassan R, Spinelli O, Oldani E, Intermesoli T, Tosi M, Peruta B, Rossi G, Borlenghi E, Pogliani EM, Terruzzi E, Fabris P, Cassibba V, Lambertenghi-Deliliers G, Cortelezzi A, Bosi A, Gianfaldoni G, Ciceri F, Bernardi M, Gallamini A, Mattei D, Di Bona E, Romani C, Scattolin AM, Barbui T, Rambaldi A: **Improved risk classification for risk-specific therapy based on the molecular study of minimal residual disease (MRD) in adult acute lymphoblastic leukemia (ALL).** *BLOOD* 2009, **113:** 4153-4162.

7. Patel B, Rai L, Buck G, Richards SM, Mortuza Y, Mitchell W, Gerrard G, Moorman AV, Duke V, Hoffbrand AV, Fielding AK, Goldstone AH, Foroni L: **Minimal residual disease is a significant predictor of treatment failure in non T-lineage adult acute lymphoblastic leukaemia: final results of the international trial UKALL XII/ECOG2993.** *Br J Haematol* 2010, **148:** 80-89.

8. Bruggemann M, Schrauder A, Raff T, Pfeifer H, Dworzak M, Ottmann OG, Asnafi V, Baruchel A, Bassan R, Benoit Y, Biondi A, Cave H, Dombret H, Fielding AK, Foa R, Gokbuget N, Goldstone AH, Goulden N, Henze G, Hoelzer D, Janka-Schaub GE, Macintyre EA, Pieters R, Rambaldi A, Ribera JM, Schmiegelow K, Spinelli O, Stary J, von Stackelberg A, Kneba M, Schrappe M, van Dongen JJ: **Standardized MRD quantification in European ALL trials: proceedings of the Second International Symposium on MRD assessment in Kiel, Germany, 18-20 September 2008.** *Leukemia* 2010, **24:** 521-535.

9. Campana D: **Minimal residual disease in acute lymphoblastic leukemia.** *Hematology Am Soc Hematol Educ Program* 2010, **2010:** 7-12.

10. van der Velden VH, Cazzaniga G, Schrauder A, Hancock J, Bader P, Panzer-Grumayer ER, Flohr T, Sutton R, Cave H, Madsen HO, Cayuela JM, Trka J, Eckert C, Foroni L, Zur Stadt U, Beldjord K, Raff T, van der Schoot CE, van Dongen JJ: **Analysis of minimal residual disease by Ig/TCR gene rearrangements: guidelines for interpretation of real-time quantitative PCR data.** *Leukemia* 2007, **21:** 604-611.

11. van der Velden VH, Wijkhuijs JM, van Dongen JJ: **Non-specific amplification of patient-specific Ig/TCR gene rearrangements depends on the time point during therapy: implications for minimal residual disease monitoring.** *Leukemia* 2008, **22:** 641-644.

12. Cazzaniga G, Valsecchi MG, Gaipa G, Conter V, Biondi A: **Defining the correct role of minimal residual disease tests in the management of acute lymphoblastic leukaemia.** *Br J Haematol* 2011, **155:** 45-52.

13. Liotta LA, Ferrari M, Petricoin E: **Clinical proteomics: written in blood.** *Nature* 2003, **425:** 905.

14. Liotta LA, Petricoin EF: **Serum peptidome for cancer detection: spinning biologic trash into diagnostic gold.** *J Clin Invest* 2006, **116:** 26-30.

15. Petricoin EF, Ardekani AM, Hitt BA, Levine PJ, Fusaro VA, Steinberg SM, Mills GB, Simone C, Fishman DA, Kohn EC, Liotta LA: **Use of proteomic patterns in serum to identify ovarian cancer.** *Lancet* 2002, **359:** 572-577.

16. Karpova MA, Moshkovskii SA, Toropygin IY, Archakov AI: **Cancer-specific MALDI-TOF profiles of blood serum and plasma: biological meaning and perspectives.** *J Proteomics* 2010, **73:** 537-551.

17. Albitar M, Potts SJ, Giles FJ, O'Brien S, Keating M, Thomas D, Clarke C, Jilani I, Aguilar C, Estey E, Kantarjian H: **Proteomic-based prediction of clinical behavior in adult acute lymphoblastic leukemia.** *Cancer* 2006, **106:** 1587-1594.

18. Shi L, Zhang J, Wu P, Feng K, Li J, Xie Z, Xue P, Cai T, Cui Z, Chen X, Hou J, Yang F: **Discovery and identification of potential biomarkers of pediatric acute lymphoblastic leukemia.** *Proteome Sci* 2009, **7:** 7.

19. Cheng AJ, Chen LC, Chien KY, Chen YJ, Chang JT, Wang HM, Liao CT, Chen IH: **Oral cancer plasma tumor marker identified with bead-based affinity-fractionated proteomic technology.** *Clin Chem* 2005, **51:** 2236-2244.

20. He J, Zeng ZC, Xiang ZL, Yang P: **Mass spectrometry-based serum peptide profiling in hepatocellular carcinoma with bone metastasis.** *World J Gastroenterol* 2014, **20:** 3025-3032.

21. Wang J, Wang X, Lin S, Chen C, Wang C, Ma Q, Jiang B: **Identification of kininogen-1 as a serum biomarker for the early detection of advanced colorectal adenoma and colorectal cancer.** *PLoS One* 2013, **8:** e70519.

22. Fan NJ, Li K, Liu QY, Wang XL, Hu L, Li JT, Gao CF: **Identification of tubulin beta chain, thymosin beta-4-like protein 3, and cytochrome b-c(1) complex subunit 1 as serological diagnostic biomarkers of gastric cancer.** *Clin Biochem* 2013, **46:** 1578-1584.

23. Fan NJ, Gao CF, Wang XL: **Tubulin beta chain, filamin A alpha isoform 1, and cytochrome b-c1 complex subunit 1 as serological diagnostic biomarkers of esophageal squamous cell carcinoma: a proteomics study.** *OMICS* 2013, **17:** 215-223.

24. Du J, Yang SY, Lin XL, Shang WL, Zhang W, Huo SF, Bu LN, Zhou B, Nan YD, Zheng HD, Liu YF: **Biomarker discovery and identification from non-small cell lung cancer sera.** *Front Biosci (Elite Ed)* 2011, **3:** 1-10.

25. Bai J, He A, Zhang W, Huang C, Yang J, Yang Y, Wang J, Zhang Y: **Potential biomarkers for adult acute myeloid leukemia minimal residual disease assessment searched by serum peptidome profiling.** *Proteome Sci* 2013, **11:** 39.

26. He A, Bai J, Huang C, Yang J, Zhang W, Wang J, Yang Y, Zhang P, Zhou F: **Detection of serum tumor markers in multiple myeloma using the CLINPROT system.** *Int J Hematol* 2012, **95:** 668-674.

27. Vermeulen R, Lan Q, Zhang L, Gunn L, McCarthy D, Woodbury RL, McGuire M, Podust VN, Li G, Chatterjee N, Mu R, Yin S, Rothman N, Smith MT: **Decreased levels of CXC-chemokines in serum of benzene-exposed workers identified by array-based proteomics.** *Proc Natl Acad Sci U S A* 2005, **102:** 17041-17046.

28. Hortin GL: **The MALDI-TOF mass spectrometric view of the plasma proteome and peptidome.** *Clin Chem* 2006, **52:** 1223-1237.

29. Qiu F, Liu HY, Zhang XJ, Tian YP: **Optimization of magnetic beads for maldi-TOF MS analysis.** *Front Biosci (Landmark Ed)* 2009, **14:** 3712-3723.

30. Bai Y, Zhang H, Sun X, Sun C, Ren L: **Biomarker identification and pathway analysis by serum metabolomics of childhood acute lymphoblastic leukemia.** *Clin Chim Acta* 2014, **436C:** 207-216.

31. Bannur Z, Teh LK, Hennesy T, Rosli WR, Mohamad N, Nasir A, Ankathil R, Zakaria ZA, Baba A, Salleh MZ: **The differential metabolite profiles of acute lymphoblastic leukaemic patients treated with 6-mercaptopurine using untargeted metabolomics approach.** *Clin Biochem* 2014, **47:** 427-431.

32. Tiziani S, Kang Y, Harjanto R, Axelrod J, Piermarocchi C, Roberts W, Paternostro G: **Metabolomics of the tumor microenvironment in pediatric acute lymphoblastic leukemia.** *PLoS One* 2013, **8:** e82859.

33. Guo Q, Zhang B, Dong X, Xie Q, Guo E, Huang H, Wu Y: **Elevated levels of plasma fibrinogen in patients with pancreatic cancer: possible role of a distant metastasis predictor.** *Pancreas* 2009, **38:** e75-79.

34. Du J, Zheng JH, Chen XS, Yang Q, Zhang YH, Zhou L, Yao X: **High preoperative plasma fibrinogen is an independent predictor of distant metastasis and poor prognosis in renal cell carcinoma.** *Int J Clin Oncol* 2013, **18:** 517-523.

35. Ghezzi F, Cromi A, Siesto G, Giudici S, Serati M, Formenti G, Franchi M: **Prognostic significance of preoperative plasma fibrinogen in endometrial cancer.** *Gynecol Oncol* 2010, **119:** 309-313.

36. Seebacher V, Polterauer S, Grimm C, Husslein H, Leipold H, Hefler-Frischmuth K, Tempfer C, Reinthaller A, Hefler L: **The prognostic value of plasma fibrinogen levels in patients with endometrial cancer: a multi-centre trial.** *Br J Cancer* 2010, **102:** 952-956.

37. Polterauer S, Seebacher V, Hefler-Frischmuth K, Grimm C, Heinze G, Tempfer C, Reinthaller A, Hefler L: **Fibrinogen plasma levels are an independent prognostic parameter in patients with cervical cancer.** *Am J Obstet Gynecol* 2009, **200:** 647 e641-647.

38. Takeuchi H, Ikeuchi S, Kitagawa Y, Shimada A, Oishi T, Isobe Y, Kubochi K, Kitajima M, Matsumoto S: **Pretreatment plasma fibrinogen level correlates with tumor progression and metastasis in patients with squamous cell carcinoma of the esophagus.** *J Gastroenterol Hepatol* 2007, **22:** 2222-2227.

39. Tao YL, Li Y, Gao J, Liu ZG, Tu ZW, Li G, Xu BQ, Niu DL, Jiang CB, Yi W, Li ZQ, Li J, Wang YM, Cheng ZB, Liu QD, Bai L, Zhang C, Zhang JY, Zeng MS, Xia YF: **Identifying FGA peptides as nasopharyngeal carcinoma-associated biomarkers by magnetic beads.** *J Cell Biochem* 2012, **113:** 2268-2278.

40. Villanueva J, Shaffer DR, Philip J, Chaparro CA, Erdjument-Bromage H, Olshen AB, Fleisher M, Lilja H, Brogi E, Boyd J, Sanchez-Carbayo M, Holland EC, Cordon-Cardo C, Scher HI, Tempst P: **Differential exoprotease activities confer tumor-specific serum peptidome patterns.** *J Clin Invest* 2006, **116:** 271-284.

41. Maciag AE, Holland RJ, Kim Y, Kumari V, Luthers CE, Sehareen WS, Biswas D, Morris NL, Ji X, Anderson LM, Saavedra JE, Keefer LK: **Nitric Oxide (NO) Releasing Poly ADP-ribose Polymerase 1 (PARP-1) Inhibitors Targeted to Glutathione S-Transferase P1-Overexpressing Cancer Cells.** *J Med Chem* 2014, **57:** 2292-2302.

42. Stella F, Weich N, Panero J, Fantl DB, Schutz N, Fundia AF, Slavutsky I: **Glutathione S-transferase P1 mRNA expression in plasma cell disorders and its correlation with polymorphic variants and clinical outcome.** *Cancer Epidemiol* 2013, **37:** 671-674.

43. Tew KD, Manevich Y, Grek C, Xiong Y, Uys J, Townsend DM: **The role of glutathione S-transferase P in signaling pathways and S-glutathionylation in cancer.** *Free Radic Biol Med* 2011, **51:** 299-313.

44. Huang GZ, Shan W, Zeng L, Huang LG: **The GSTP1 A1578G polymorphism and the risk of childhood acute lymphoblastic leukemia: results from an updated meta-analysis.** *Genet Mol Res* 2013, **12:** 2481-2491.

45. Wei B, Zhou Y, Xu Z, Ruan J, Cheng H, Zhu M, Hu Q, Jin K, Yan Z, Zhou D, Xuan F, Zhou H, Wang Z, Huang X, Wang Q: **GSTP1 Ile105Val polymorphism and prostate cancer risk: evidence from a meta-analysis.** *PLoS One* 2013, **8:** e71640.

46. Xie P, Liang Y, Liang G, Liu B: **Association between GSTP1 Ile105Val polymorphism and glioma risk: a systematic review and meta-analysis.** *Tumour Biol* 2014, **35:** 493-499.

47. Tan Z, Feng M, Luo Y, Sun C, Fan Z, Tan Y, Fu B, Lang J: **GSTP1 Ile105Val polymorphism and colorectal cancer risk: an updated analysis.** *Gene* 2013, **527:** 275-282.

48. Jin P, Xie J, Zhu X, Zhou C, Ding X, Yang L: **shRNA-mediated GSTP1 gene silencing enhances androgen-independent cell line DU145 chemosensitivity.** *Int Urol Nephrol* 2013.

49. Mutallip M, Nohata N, Hanazawa T, Kikkawa N, Horiguchi S, Fujimura L, Kawakami K, Chiyomaru T, Enokida H, Nakagawa M, Okamoto Y, Seki N: **Glutathione S-transferase P1 (GSTP1) suppresses cell apoptosis and its regulation by miR-133alpha in head and neck squamous cell carcinoma (HNSCC).** *Int J Mol Med* 2011, **27:** 345-352.

50. Gatedee J, Pakakassama S, Muangman S, Pongstaporn W: **Glutathione S-transferase P1 genotypes, genetic susceptibility and outcome of therapy in thai childhood acute lymphoblastic leukemia.** *Asian Pac J Cancer Prev* 2007, **8:** 294-296.

51. Dunna NR, Vuree S, Kagita S, Surekha D, Digumarti R, Rajappa S, Satti V: **Association of GSTP1 gene (I105V) polymorphism with acute leukaemia.** *J Genet* 2012, **91:** e60-63.

52. Aivado M, Spentzos D, Germing U, Alterovitz G, Meng XY, Grall F, Giagounidis AA, Klement G, Steidl U, Otu HH, Czibere A, Prall WC, Iking-Konert C, Shayne M, Ramoni MF, Gattermann N, Haas R, Mitsiades CS, Fung ET, Libermann TA: **Serum proteome profiling detects myelodysplastic syndromes and identifies CXC chemokine ligands 4 and 7 as markers for advanced disease.** *Proc Natl Acad Sci U S A* 2007, **104:** 1307-1312.

53. Kim JY, Song HJ, Lim HJ, Shin MG, Kim JS, Kim HJ, Kim BY, Lee SW: **Platelet factor-4 is an indicator of blood count recovery in acute myeloid leukemia patients in complete remission.** *Mol Cell Proteomics* 2008, **7:** 431-441.

54. Bassan R, Gatta G, Tondini C, Willemze R: **Adult acute lymphoblastic leukaemia.** *Crit Rev Oncol Hematol* 2004, **50:** 223-261.

55. Eckert C, Flohr T, Koehler R, Hagedorn N, Moericke A, Stanulla M, Kirschner-Schwabe R, Cario G, Stackelberg A, Bartram CR, Henze G, Schrappe M, Schrauder A: **Very early/early relapses of acute lymphoblastic leukemia show unexpected changes of clonal markers and high heterogeneity in response to initial and relapse treatment.** *Leukemia* 2011, **25:** 1305-1313.

56. Zhao J, He A, Zhang W, Meng X, Gu L: **Quantitative assessment of MLAA-34 expression in diagnosis and prognosis of acute monocytic leukemia.** *Cancer Immunol Immunother* 2011, **60:** 587-597.

# Figure legends

## Figure 1 Serum peptide fingerprints and cluster analysis of newly diagnosed ALL and healthy control.

**A.** Comparison of serum peptide fingerprints between ALL patients and healthy controls showed that peak number and intensity of the two groups were completely different. **B.** Stack view of comparison between ALL patients and healthy controls.

(Red: ALL newly diagnosed group Green: Healthy control group)

## Figure 2 Relative intensities comparisons of peptides from representative samples of ND ALL and HC.

**A.** Upregulation of the peptide with MW of 2661.27Da in ALL ND group. **B.** Upregulation of the peptide with MW of 2991.46Da in ALL ND group. **C.** Downregulation of the peptide with MW of 3443.92Da in ALL ND group. **D.** Downregulation of the peptide with MW of 7764.29Da in ALL ND group. **E.** Downregulation of the peptide with MW of 9288.31 Da in ALL ND group. (ND: newly diagnosed; HC: healthy control; MW: molecular weight).

(Red: ALL newly diagnosed group Green: Healthy control group)

## Figure 3 The diagnostic capability of each peak in QC model determined by ROC curve.

**A.** Area under the curve (AUC) of the peptide with MW of 2661.27Da is 92.56%, representing higher diagnostic value. **B.**AUC of the peptide with MW of 2991.46Da is 89.22%, representing moderate diagnostic value. **C.** AUC of the peptide with MW of 3443.92Da is 91%, representing higher diagnostic value. **D.** AUC of the peptide with MW of 7764.29Da is 86.56%, representing moderate diagnostic value. **E.** AUC of the peptide with MW of 9288.31Da is 90.22%, representing higher diagnostic value.

## Figure 4 MS/MS map of peptide with MW of 2661.27Da.

**A.** The enlarged picture of peptide with MW of 2661.27Da. **B.** The b and y ions spectra are used to identify the peptide with MW of 2661.27Da. **C.** The sequence of the peptide with MW of 2661.27Da.

## Figure 5 MS/MS map of peptide with MW of 2991.46Da.

**A.** The enlarged picture of peptide with MW of 2991.46Da. **B.** The b and y ions spectra are used to identify the peptide with MW of 2991.46Da. **C.** The sequence of the peptide with MW of 2991.46Da.

## Figure 6 MS/MS map of peptide with MW of 3443.92Da.

**A.** The enlarged picture of peptide with MW of 3443.92Da. **B.** The b and y ions spectra are used to identify the peptide with MW of 3443.92Da. **C.** The sequence of the peptide with MW of 3443.92Da.

## Figure 7 MS/MS map of peptide with MW of 7764.29Da.

**A.** The enlarged picture of peptide with MW of 7764.29Da. **B.** The b and y ions spectra are used to identify the peptide with MW of 7764.29Da. **C.** The sequence of the peptide with MW of 7764.29Da.

## Figure 8 Impact of the relative intensities of five peptides on relapse.

**A.** No correlation was found between relative intensity of peptide m/z 2661.27 and ALL relapse time after initial diagnosis. **B.** Newly diagnosed ALL patients with higher relative intensity of peptide m/z 2991.46 relapsed earlier than those with lower relative intensity. **C.** No correlation was found between relative intensity of peptide m/z 3443.92 and ALL relapse time after initial diagnosis. **D.** Newly diagnosed ALL patients with lower relative intensity of peptide m/z 7764.29 had a significantly earlier relapse. **E.** Lower relative intensity of peptide m/z 9288.31 in newly diagnosed ALL patients was associated with an earlier relapse.

## Figure 9 Validation of proteins by immunoblotting.

**A.** Levels of the FGA and GSTP1 protein differ among the leukemia cells in distinct ALL groups and normal cells. Weak PF4 immunoreactive bands are seen in newly diagnosed and refractory & relapsed ALL cases. **B.** Densitometry comparison of FGA protein relative to β-actin as determined by western blot analysis in figure A. **C.** Densitometry comparison of GSTP1 protein relative to β-actin as determined by western blot analysis in figure A. **D.** Densitometry comparison of PF4 protein relative to β-actin as determined by western blot analysis in figure A.

(FGA: fibrinogen alpha chain; GSTP1: glutathione S-transferase P1; PF4: platelet factor 4; ALL- CR: ALL complete remission; ALL-ND: ALL newly diagnosed; ALL-RR: ALL refractory & relapsed)

## Figure 10 Correlation analyses between serum PF4/CTAP-III contents and platelet counts in newly diagnosed ALL.

Linear regression analyses show that no correlations are found between PF4 (A)/CTAP-III (B) contents and platelet counts.

## Suppl. Figure 1 ROC curves of the relative intensities of peptides differentiating patients with CR from RR.

**A.** Area under the curve (AUC) of the peptide with MW of 2661.27Da is 92.3%, representing higher diagnostic value for distinguishing CR from RR patients. **B.** AUC of the peptide with MW of 2991.46Da is 100%, representing higher diagnostic value for distinguishing CR from RR patients. **C.** AUC of the peptide with MW of 3443.92Da is 91%, representing higher diagnostic value for distinguishing CR from RR patients. **D.** AUC of the peptide with MW of 7764.29Da is 89.5%, representing moderate diagnostic value for distinguishing CR from RR patients. **E.** AUC of the peptide with MW of 9288.31Da is 98.7%, representing higher diagnostic value for distinguishing CR from RR patients.

## Suppl. Figure 2 Correlation analyses between contents of serum peptides and platelet/WBC counts in healthy control group.

**A.** Correlation coefficient of FGA contents and platelet counts is 0.044 (p=0.787). **B.** Correlation coefficient of FGA contents and WBC counts is 0.027 (p=0.869). **C.** Correlation coefficient of GSTP1 contents and platelet counts is 0.191 (p=0.238). **D.** Correlation coefficient of GSTP1 contents and WBC counts is 0.079 (p=0.630). **E.** Correlation coefficient of PF4 contents and platelet counts is 0.171 (p=0.292). **F.** Correlation coefficient of PF4 contents and WBC counts is 0.139 (p=0.394). **G.** Correlation coefficient of CTAP-III contents and platelet counts is 0.033 (p=0.838). **H.** Correlation coefficient of CTAP-III contents and WBC counts is 0.070 (p=0.669). (FGA: fibrinogen alpha chain; GSTP1: glutathione S-transferase P1; PF4: platelet factor 4; CTAP-III: connective tissue active peptide III; WBC: white blood cell)

## Suppl. Figure 3 Correlation analyses between contents of serum peptides and platelet/WBC counts in ALL complete remission group.

**A.** Correlation coefficient of FGA contents and platelet counts is 0.097 (p=0.552). **B.** Correlation coefficient of FGA contents and WBC counts is 0.097 (p=0.552). **C.** Correlation coefficient of GSTP1 contents and platelet counts is 0.126 (p=0.440). **D.** Correlation coefficient of GSTP1 contents and WBC counts is 0.185 (p=0.254). **E.** Correlation coefficient of PF4 contents and platelet counts is 0.157 (p=0.333). **F.** Correlation coefficient of PF4 contents and WBC counts is 0.236 (p=0.143). **G.** Correlation coefficient of CTAP-III contents and platelet counts is 0.146 (p=0.369). **H.** Correlation coefficient of CTAP-III contents and WBC counts is 0.128 (p=0.431).

(FGA: fibrinogen alpha chain; GSTP1: glutathione S-transferase P1; PF4: platelet factor 4; CTAP-III: connective tissue active peptide III; WBC: white blood cell)

## Suppl. Figure 4 Correlation analyses between contents of serum peptides and platelet/WBC counts in ALL refractory&relapsed group.

**A.** Correlation coefficient of FGA contents and platelet counts is 0.043 (p=0.792). **B.** Correlation coefficient of FGA contents and WBC counts is 0.057 (p=0.728). **C.** Correlation coefficient of GSTP1 contents and platelet counts is 0.086 (p=0.596). **D.** Correlation coefficient of GSTP1 contents and WBC counts is 0.054 (p=0.741).**E.** Correlation coefficient of PF4 contents and platelet counts was 0.079 (p=0.630). **F.** Correlation coefficient of PF4 contents and WBC counts is 0.102 (p=0.529). **G.** Correlation coefficient of CTAP-III contents and platelet counts is 0.079 (p=0.627). **H.** Correlation coefficient of CTAP-III contents and WBC counts is 0.012 (p=0.941).

(FGA: fibrinogen alpha chain; GSTP1: glutathione S-transferase P1; PF4: platelet factor 4; CTAP-III: connective tissue active peptide III; WBC: white blood cell)

## Suppl. Figure 5 Correlation analyses between contents of serum peptides and platelet/WBC counts in ALL newly diagnosed group.

**A.** Correlation coefficient of FGA contents and platelet counts is 0.253 (p=0.115). **B.** Correlation coefficient of FGA contents and WBC counts is 0.244 (p=0.129). **C.** Correlation coefficient of GSTP1 contents and platelet counts is 0.207 (p=0.200). **D.** Correlation coefficient of GSTP1 contents and WBC counts is 0.118 (p=0.468). **E.** Correlation coefficient of PF4 contents and WBC counts is 0.178 (p=0.271). **F.** Correlation coefficient of CTAP-III contents and WBC counts is 0.148 (p=0.361).

(FGA: fibrinogen alpha chain; GSTP1: glutathione S-transferase P1; PF4: platelet factor 4; CTAP-III: connective tissue active peptide III; WBC: white blood cell)

## Suppl. Figure 6 ROC curves of the serum contents of proteins differentiating patients with CR from RR.

**A.** Area under the curve (AUC) of FGA is 99.6%. **B.**AUC of GSTP1 is 99.4%. **C.** AUC of PF4 is 99.8%. **D.** AUC of CATP-III is 99.8%. AUCs of the four proteins are all ≥90%, representing higher diagnostic values of the serum contents of the four proteins for distinguishing CR from RR patients.

(FGA: fibrinogen alpha chain; GSTP1: glutathione S-transferase P1; PF4: platelet factor 4; CTAP-III: connective tissue active peptide III)

# Tables

## Table 1 Different expressed peptides between newly diagnosed ALL and healthy controls

| **Index** | **Mass** | **Dave** | **PWKW** | **Ave1** | **Ave2** | **StdDev1** | **StdDev2** | **Expression Change** |
| --- | --- | --- | --- | --- | --- | --- | --- | --- |
| 61 | 9288.31 | 2.32 | < 0.000001 | 1.31 | 3.63 | 0.28 | 1.87 | **↓** |
| 27 | 3443.92 | 1.1 | < 0.000001 | 1.54 | 2.64 | 0.66 | 0.9 | **↓** |
| 41 | 4249.06 | 0.76 | 0.000007 | 1.7 | 2.46 | 0.62 | 0.49 | **↓** |
| 13 | 2661.27 | 20.95 | < 0.000001 | 28.39 | 7.44 | 22.56 | 3.69 | **↑** |
| 43 | 4645.31 | 1.74 | 0.00000373 | 1.67 | 3.41 | 1.09 | 1.75 | **↓** |
| 7 | 1945.51 | 1.06 | 0.00000114 | 2.01 | 3.06 | 0.39 | 1.12 | **↓** |
| 8 | 2093.83 | 1.15 | 0.0000791 | 3.38 | 2.23 | 1.38 | 0.44 | **↑** |
| 33 | 4072.42 | 0.4 | 0.00175 | 1.59 | 1.99 | 0.43 | 0.4 | **↓** |
| 35 | 4108.67 | 0.45 | 0.00129 | 1.66 | 2.1 | 0.53 | 0.41 | **↓** |
| 28 | 3884.26 | 0.85 | 0.000281 | 1.65 | 2.5 | 0.82 | 1.02 | **↓** |
| 4 | 1546.94 | 5.98 | 0.000942 | 10.48 | 4.5 | 8.72 | 2.57 | **↑** |
| 12 | 2644.19 | 0.86 | 0.001 | 2.93 | 2.07 | 1.23 | 0.5 | **↑** |
| 36 | 4123.33 | 0.42 | 0.00445 | 1.7 | 2.13 | 0.54 | 0.43 | **↓** |
| 62 | 9367.9 | 1.28 | 0.0108 | 0.95 | 2.23 | 0.41 | 1.2 | **↓** |
| 11 | 2561.9 | 1.28 | 0.000009 | 3.07 | 1.79 | 1.97 | 0.49 | **↑** |
| 21 | 2991.46 | 5.28 | < 0.000001 | 17.24 | 1.96 | 8.54 | 0.63 | **↑** |
| 34 | 4091.61 | 1.46 | 0.00622 | 3.16 | 4.62 | 1.55 | 1.93 | **↓** |
| 58 | 7764.29 | 1.3 | 0.00000217 | 1.39 | 2.68 | 0.7 | 1.44 | **↓** |
| 10 | 2545.82 | 0.92 | 0.00619 | 2.98 | 2.06 | 1.53 | 0.47 | **↑** |
| 46 | 5064.98 | 0.33 | 0.0374 | 1.11 | 1.44 | 0.27 | 0.53 | **↓** |
| 2 | 1466.84 | 5.01 | 0.000639 | 8.46 | 3.46 | 4.84 | 2.44 | **↑** |
| 1 | 1450.14 | 1 | 0.0163 | 4.09 | 3.1 | 1.72 | 0.68 | **↑** |
| 57 | 7025.82 | 0.69 | 0.00913 | 0.83 | 1.52 | 0.35 | 1.23 | **↓** |
| 14 | 2673.64 | 1.7 | 0.00626 | 4.04 | 2.34 | 1.22 | 0.53 | **↑** |
| 42 | 4266.78 | 1 | 0.0154 | 2.56 | 3.57 | 1.22 | 1.6 | **↓** |
| 18 | 2900.77 | 0.89 | 0.0288 | 2.62 | 1.74 | 1.71 | 0.48 | **↑** |
| 5 | 1618.2 | 3.56 | 0.0000421 | 6.76 | 3.19 | 4.43 | 1.65 | **↑** |
| 3 | 1526.98 | 1.92 | 0.0107 | 4.29 | 2.37 | 2.03 | 0.61 | **↑** |
| 38 | 4169.26 | 0.43 | 0.0146 | 2.04 | 2.46 | 0.81 | 0.54 | **↓** |
| 39 | 4194.35 | 0.71 | 0.0173 | 2.88 | 3.59 | 1.35 | 1.01 | **↓** |
| 29 | 3891.28 | 0.44 | 0.0285 | 1.83 | 0.77 | 0.86 | 0.17 | **↓** |
| 22 | 3192.79 | 0.38 | 0.00906 | 2.03 | 2.4 | 1.19 | 0.78 | **↓** |
| 25 | 3263.65 | 0.84 | 0.00446 | 2.98 | 3.82 | 1.7 | 1.75 | **↓** |

Index: peptide peak index; Mass: mass to charge ratio value; Dave: differences of average peak intensity between newly diagnosed ALL group and healthy control group; P value: p value of Wilcoxon test; Ave1: average peak intensity of newly diagnosed ALL group; Ave2: average peak intensity of healthy control group; StdDev1: standard deviation of the peak intensity average of newly diagnosed ALL group; StdDev2: standard deviation of the peak intensity average of healthy control group.

## Table 2 Identification results of peptides in QC model

| **Molecular weight** | **Amino acid sequence** | [**International Protein Index**](http://www.ebi.ac.uk/IPI/) | **Peptide name** |
| --- | --- | --- | --- |
| 2661.27Da | DEAGSEADHEGTHSTKRGHAKSRPV | IPI00021885 | fibrinogen alpha chain |
| 2991.46Da | MLLADQGQSWKEEVVTVETWQEGSLK | IPI00219757.13 | glutathione S- transferase P1 |
| 3443.92Da | HRHPDEAAFFDTASTGKTFPGFFSPMLGEFV | IPI00021885.1 | isoform 1 of fibrinogen alpha chain precursor |
| 7764.29Da | EAEEDGDLQCLCVKTTSQVRPRHITSLEVIKAGPHCPTAQLIATLKNGRKICLDLQAPLYKKIIKKLLES | IPI 00022446 | platelet factor 4 |

## Table 3 Relative intensity of peptides in the QC model in different ALL groups

| **Mass** | **Ave1±StdDev1** | **Ave2±StdDev2** | **Ave3±StdDev3** | **Ave4±StdDev4** | **n1** | **n2** | **n3** | **n4** |
| --- | --- | --- | --- | --- | --- | --- | --- | --- |
| 2661.27 | 28.39±22.56 | 7.44±3.69 | 7.76±3.48 | 31.08±23.58 | 84 | 85 | 44 | 45 |
| 2991.46 | 17.24±8.54 | 1.96±0.63 | 1.63±0.52 | 18.51±8.75 |  |  |  |  |
| 3443.92 | 1.54±0.66 | 2.64±0.9 | 2.62±0.86 | 1.23±0.58 |  |  |  |  |
| 7764.29 | 1.39±0.7 | 2.68±1.44 | 2.71±1.45 | 1.21±0.57 |  |  |  |  |
| 9288.31 | 1.31±0.28 | 3.63±1.87 | 3.9±1.66 | 1.01±0.26 |  |  |  |  |

Class1: ALL ND group; Class2: HC group; Class3: ALL CR group; Class4: ALL RR group; Mass: molecular weight; Ave: average peak intensity; Std: standard deviation; n: case number.

## Table 4 Clinical features of patients in different ALL groups before chemotherapy

| **Clinical features** | | **ALL** | | |
| --- | --- | --- | --- | --- |
|  |  | **Newly Diagnosed** | **CR** | **Refractory &Relapsed** |
| Sex | male | 50  34 | 24  20 | 26  19 |
|  | female |  |  |  |
| Age(year) | | 31(18-77) | 29(18-62) | 27(18-77) |
| WBC(×10^9^/L) | | 53(0.9-367.4) | 14.75(3.01-39.65) | 25.99(0.45-102.56) |
| Hb(g/L) | | 70(31-143) | 108(84-394) | 75(48-139) |
| PLT(×10^9^/L) | | 37(1-338) | 105(25-394) | 45(2-245) |
| Subtype | | T 14  B 70 | T 4  B 40 | T 4  B 41 |
| Chromosome Abnormality | | Ph+ 20  del(5)(p14) 2  21+mar 2 | Ph+ 6  21+mar 1 | Ph+ 10  del(5)(p14) 1 |
| Molecular Genetics Abnormality | | bcr-abl 20 | bcr-abl 6 | bcr-abl 10 |
| Sternal tenderness | | 54/84 | 20/44 | 27/45 |
| Lymphadenectasis | | 50/84 | 25/44 | 26/45 |
| Splenohepatomegalia | | 45/84 | 21/44 | 23/45 |
| Curative effect | | 69/84 | 44/44 | 36/45 |

This table shows clinical features of ALL patients in different groups at the time of diagnosis. WBC: white blood cell; Hb: hemoglobin; PLT: platelet. Curative effect refers to achieving complete remission after 2 courses of standard chemotherapy.

## Suppl. Table RI of peptides with respect to the clinical characteristics at diagnosis

|  | | **Mass** | | | | |
| --- | --- | --- | --- | --- | --- | --- |
|  |  | **2661.27** | **2991.46** | **3443.92** | **7764.29** | **9288.31** |
| **Subtype** | T-ALL  B-ALL  p value | 28.68±22.58  28.33±22.53  0.76 | 17.35±8.55  17.22±8.51  0.78 | 1.58±0.67  1.53±0.64  0.85 | 1.42±0.73  1.38±0.67  0.68 | 1.35±0.29  1.30±0.26  0.79 |
| **Gender** | Female  Male  p value | 29.15±22.71  27.87±22.47  0.78 | 17.48±8.58  17.08±8.47  0.81 | 1.56±0.65  1.53±0.62  0.87 | 1.41±0.72  1.38±0.68  0.65 | 1.33±0.28  1.30±0.25  0.78 |
| **Age** | ≥60y  <60y  p value | 28.72±22.61  28.23±22.52  0.75 | 17.31±8.53  17.21±8.50  0.87 | 1.57±0.66  1.53±0.62  0.83 | 1.42±0.72  1.38±0.68  0.69 | 1.36±0.29  1.29±0.24  0.76 |


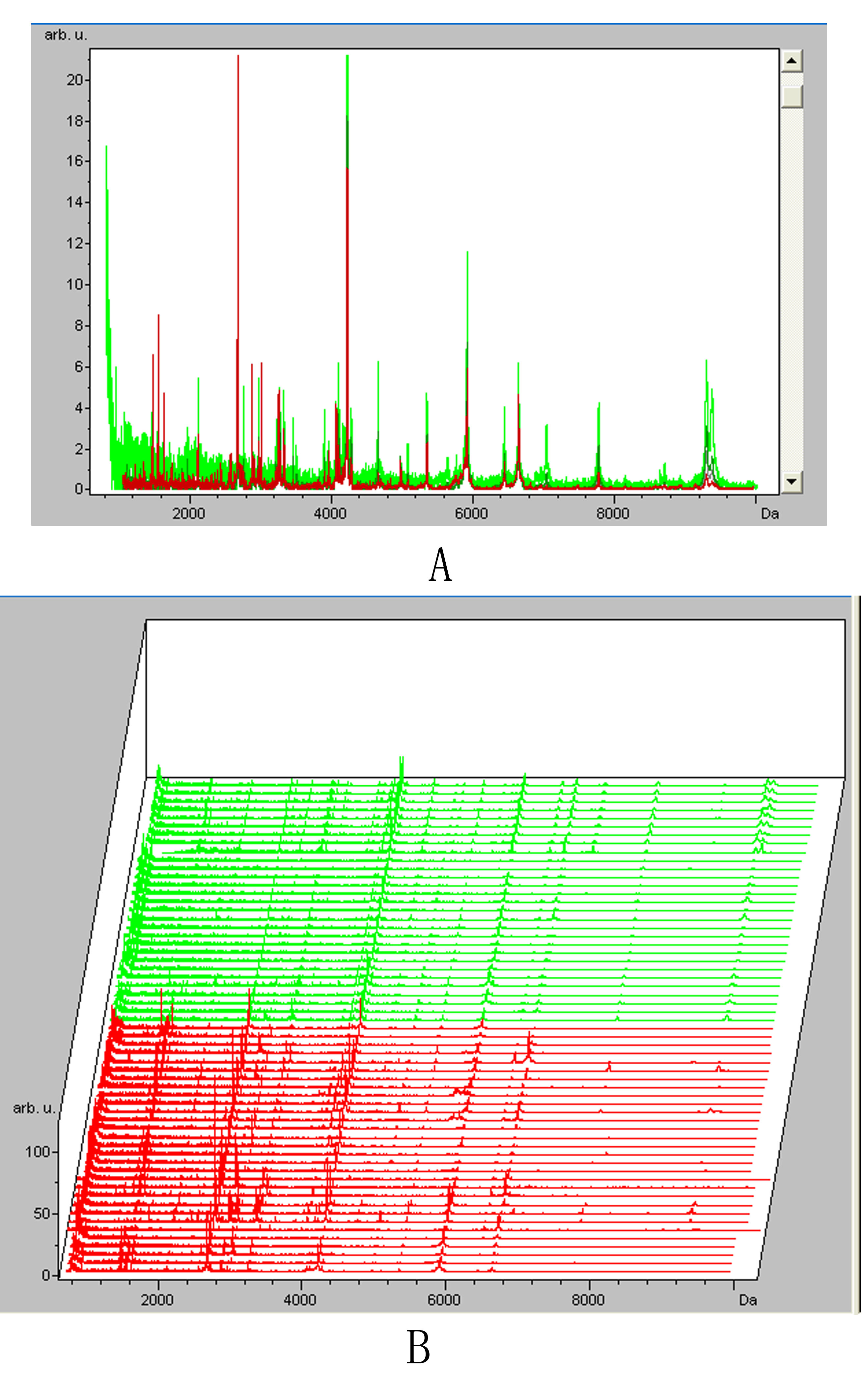


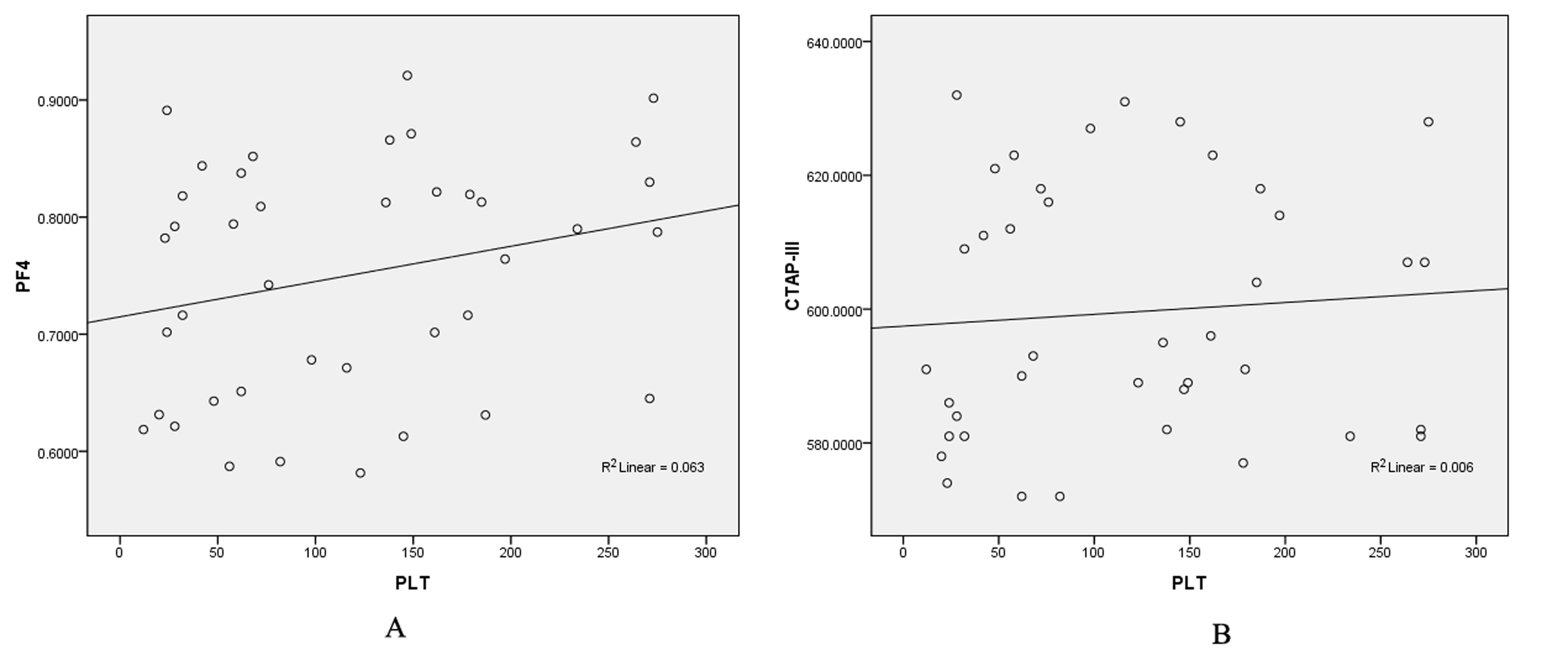





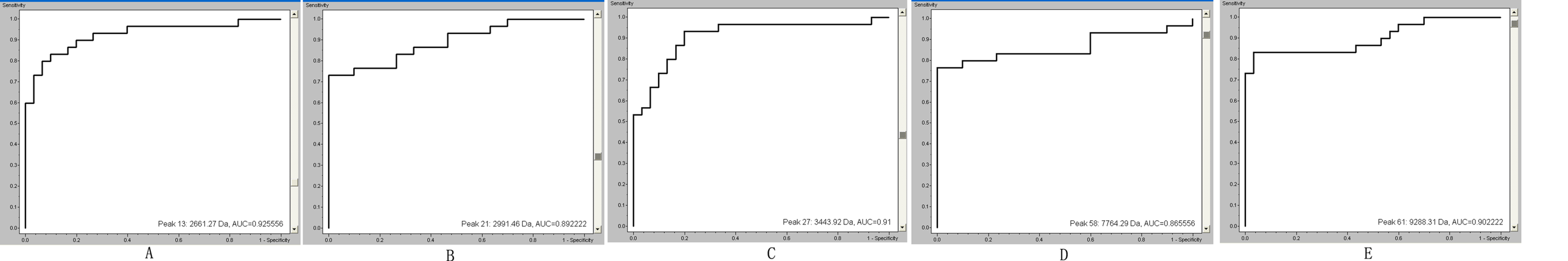


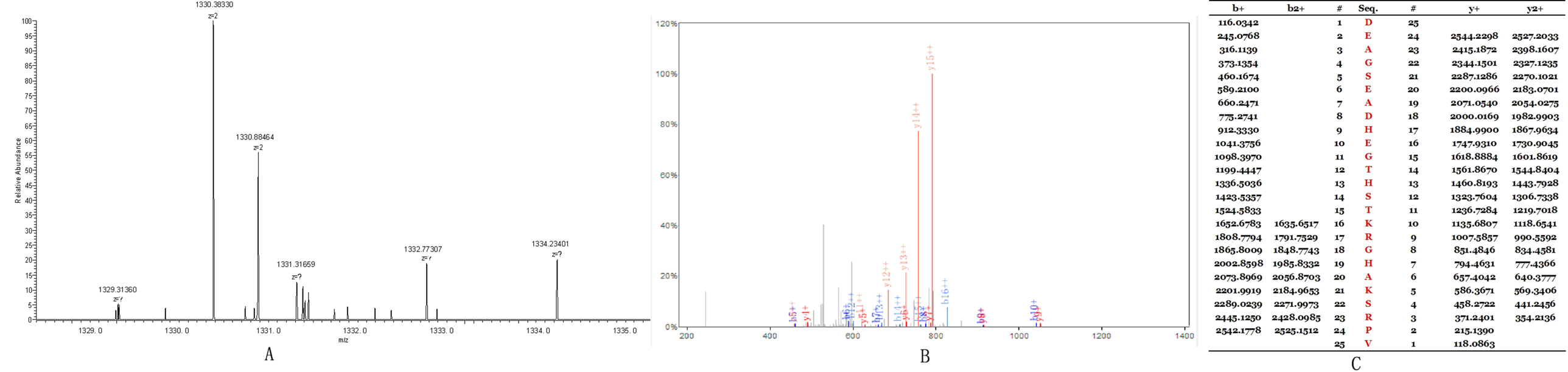


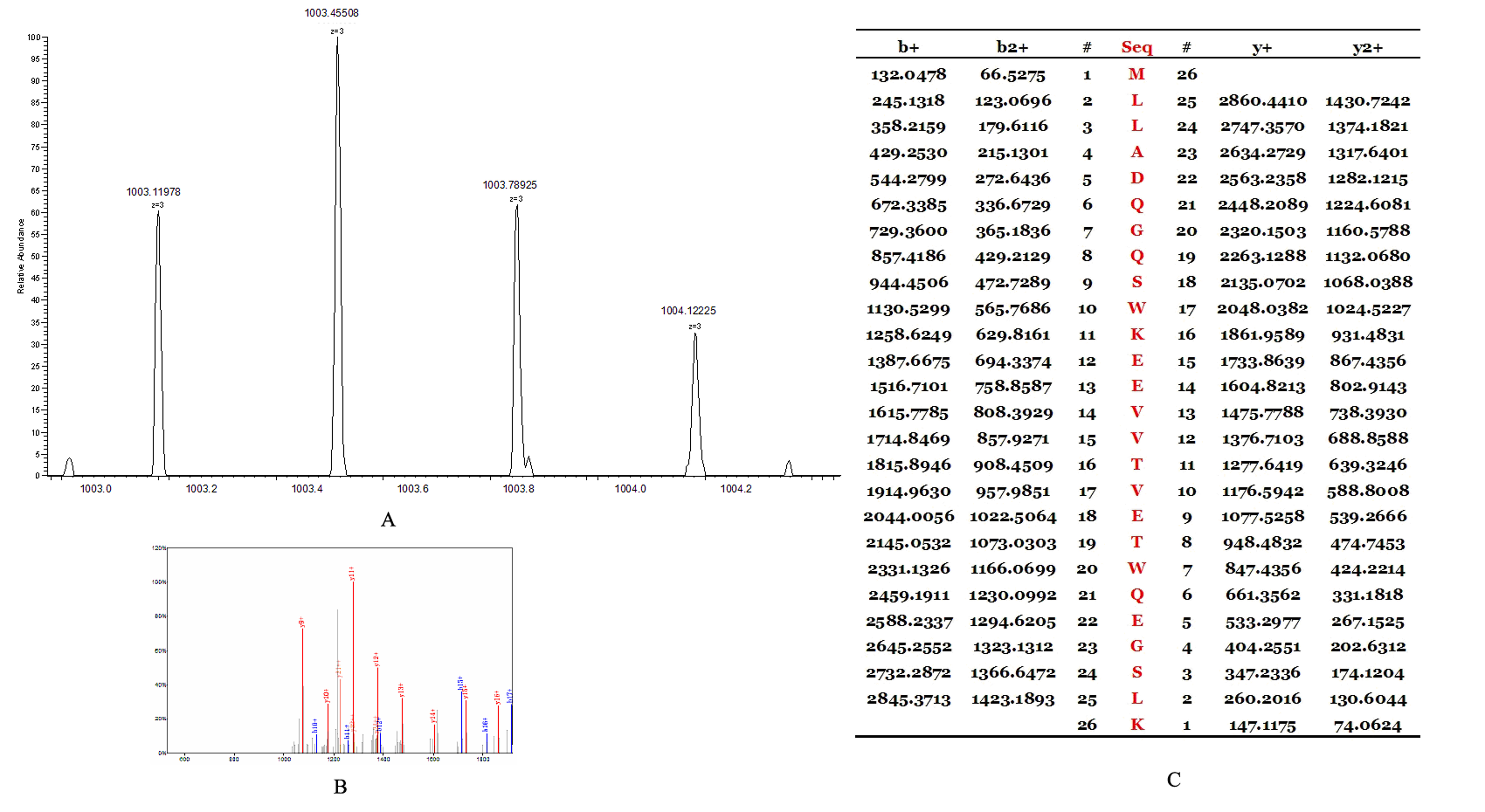


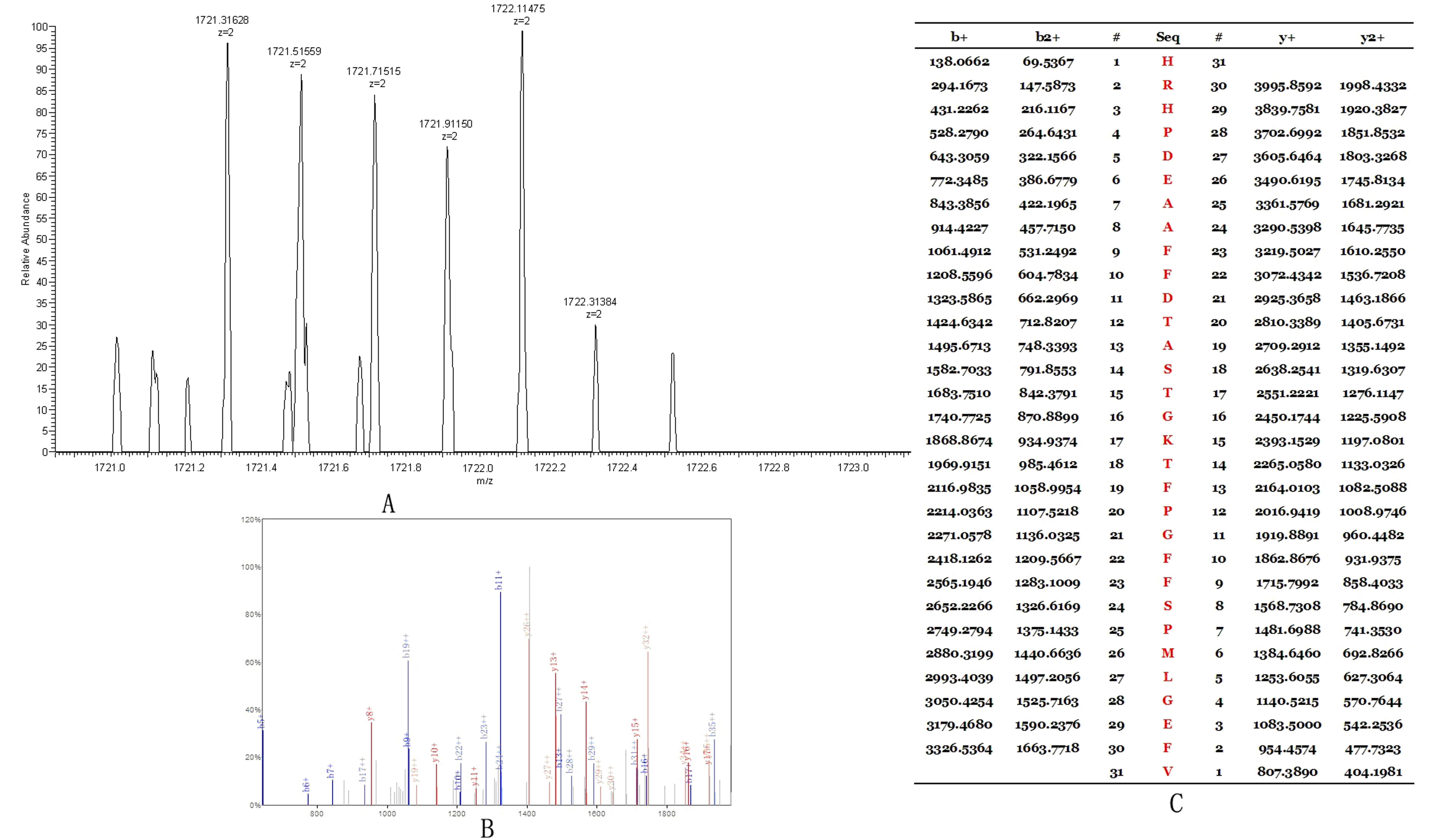


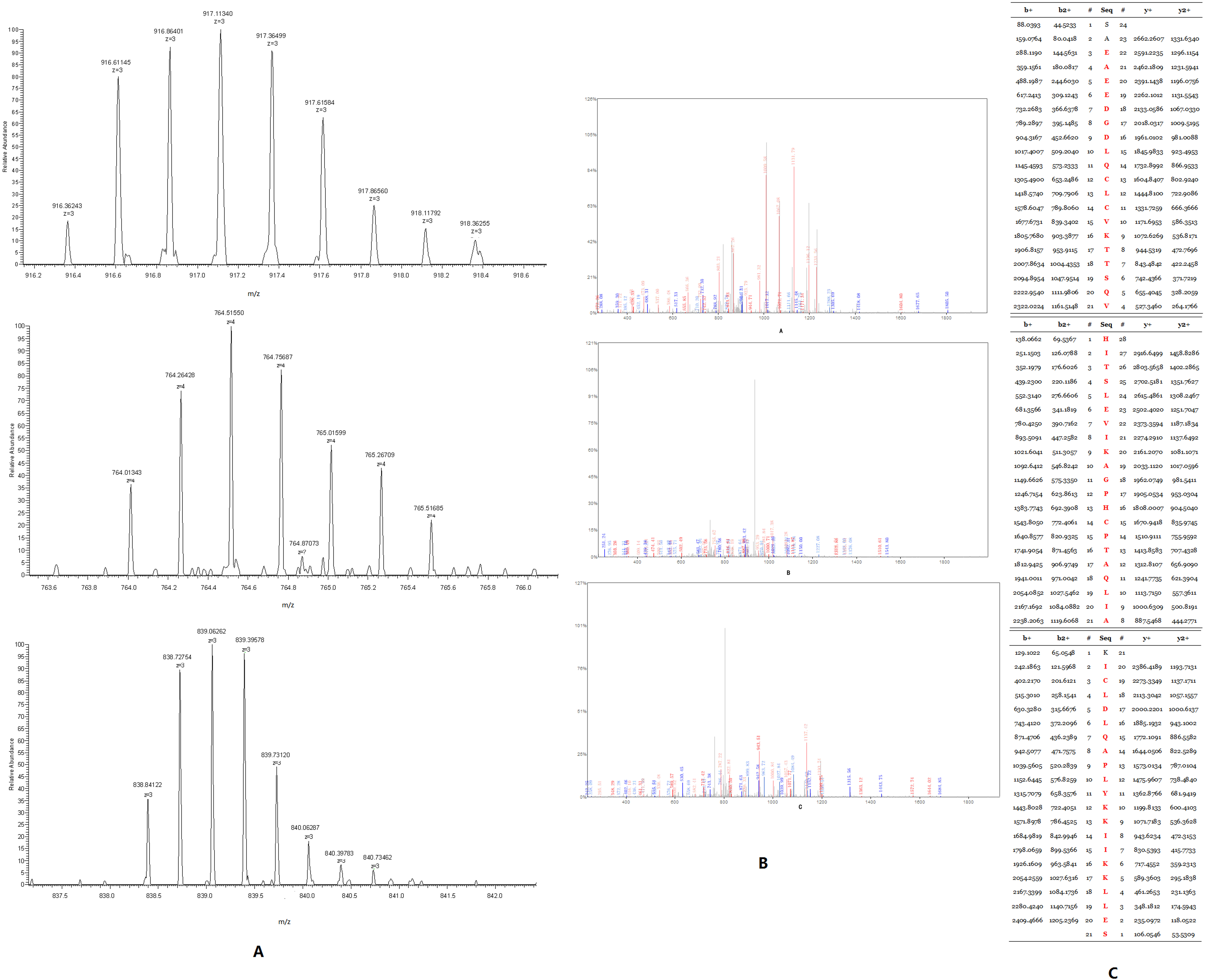


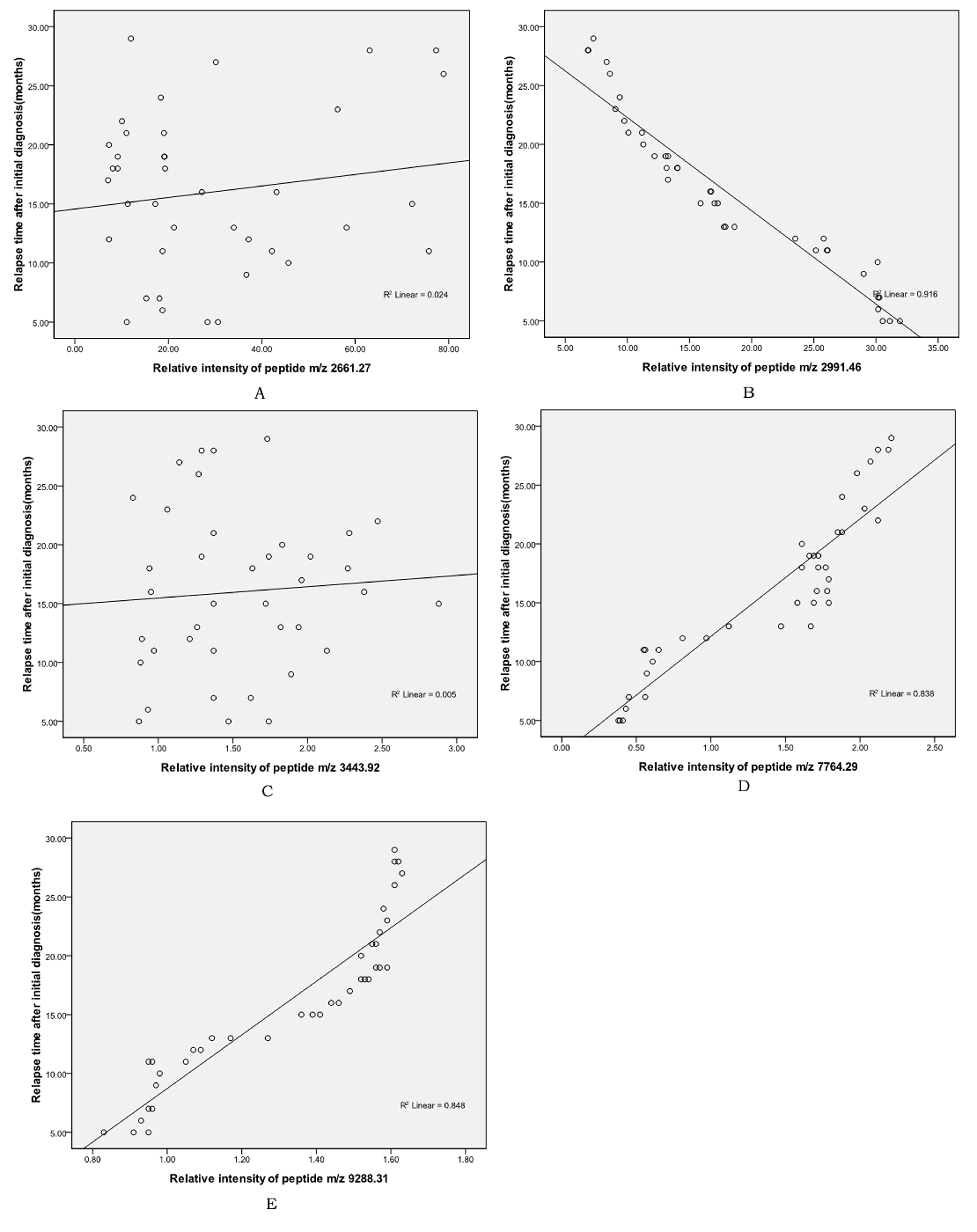


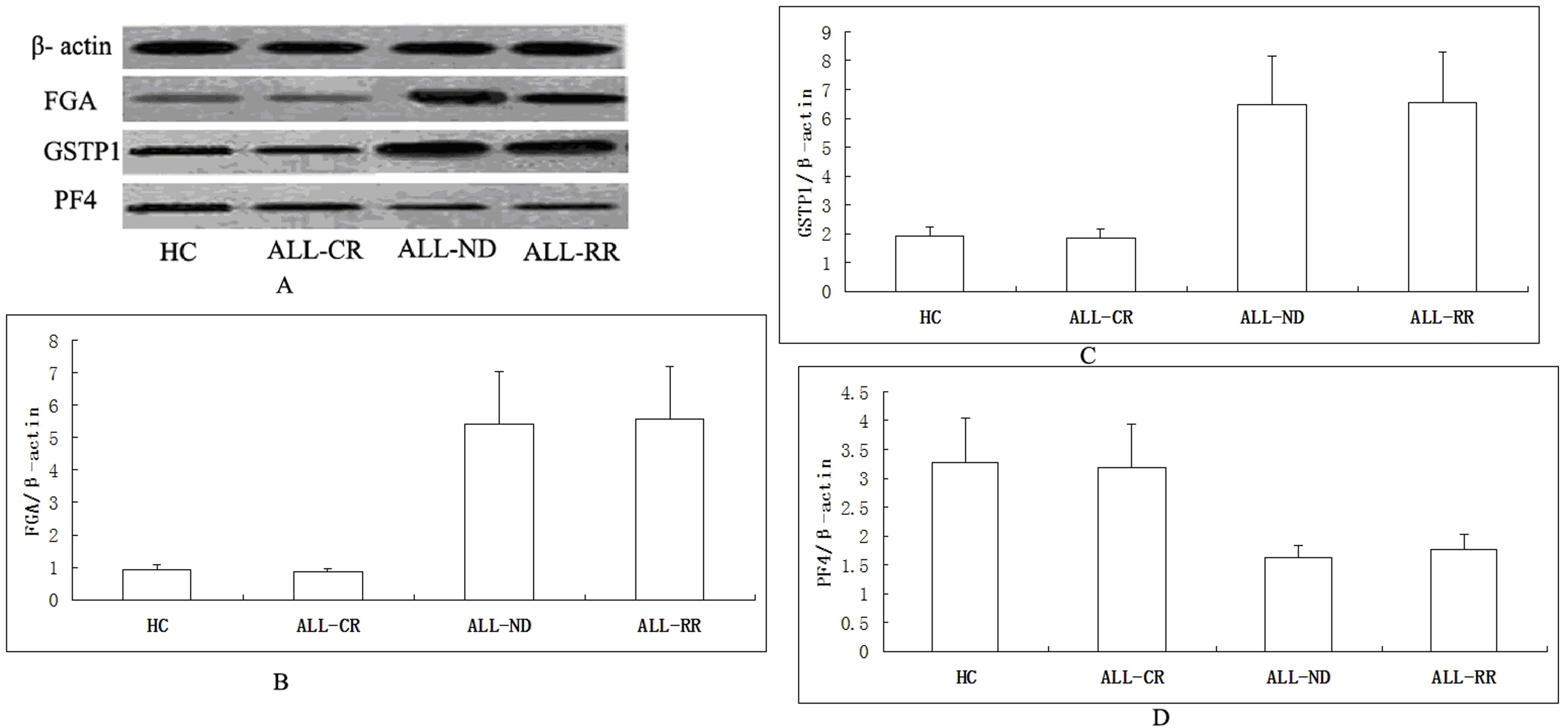

Supplement: Additional file 2: Table S1. — RI of peptides with respect to the clinical characteristics at diagnosis. [file 12953_2014_49_MOESM2_ESM.doc]
